# Multispectral imaging of formalin-fixed tissue predicts ability to generate tumor-infiltrating lymphocytes from melanoma

**DOI:** 10.1186/s40425-015-0091-z

**Published:** 2015-10-20

**Authors:** Zipei Feng, Sachin Puri, Tarsem Moudgil, William Wood, Clifford C. Hoyt, Chichung Wang, Walter J. Urba, Brendan D. Curti, Carlo B. Bifulco, Bernard A. Fox

**Affiliations:** Robert W. Franz Cancer Research Center, Earle A. Chiles Research Institute, Providence Cancer Center, Providence Portland Medical Center, 4805 NE Glisan St, Portland, OR 97213 USA; Departments of Cancer Biology, Oregon Health & Science University, Portland, OR USA; PerkinElmer, Waltham, MA USA; Department of, Providence Portland Regional Laboratory, Portland, OR USA; Department of Molecular Microbiology and Immunology, Oregon Health & Science University, Portland, OR USA; UbiVac, Portland, OR USA

**Keywords:** Tumor-infiltrating lymphocytes (TIL), multispectral imaging, Adoptive T cell therapy (ACT), Immunotherapy, Melanoma, Immunoprofiling, Immunoscore, Immunotherapy biomarker

## Abstract

**Background:**

Adoptive T cell therapy (ACT) has shown great promise in melanoma, with over 50 % response rate in patients where autologous tumor-reactive tumor-infiltrating lymphocytes (TIL) can be cultured and expanded. A major limitation of ACT is the inability to generate or expand autologous tumor-reactive TIL in 25–45 % of patients tested. Methods that successfully identify tumors that are not suitable for TIL generation by standard methods would eliminate the costs of fruitless expansion and enable these patients to receive alternate therapy immediately.

**Methods:**

Multispectral fluorescent immunohistochemistry with a panel including CD3, CD8, FoxP3, CD163, PD-L1 was used to analyze the tumor microenvironment in 17 patients with melanoma among our 36-patient cohort to predict successful TIL generation. Additionally, we compared tumor fragments and enzymatic digestion of tumor samples for efficiency in generating tumor-reactive TIL.

**Results:**

Tumor-reactive TIL were generated from 21/36 (58 %) of melanomas and for 12/13 (92 %) tumors where both enzymatic and fragment methods were compared. TIL generation was successful in 10/13 enzymatic preparations and in 10/13 fragment cultures; combination of both methods resulted in successful generation of autologous tumor-reactive TIL in 12/13 patients. In 17 patients for whom tissue blocks were available, IHC analysis identified that while the presence of CD8^+^ T cells alone was insufficient to predict successful TIL generation, the CD8^+^ to FoxP3^+^ ratio was predictive with a positive-predictive value (PPV) of 91 % and negative-predictive value (NPV) of 86 %. Incorporation of CD163+ macrophage numbers and CD8:PD-L1 ratio did not improve the PPV. However, the NPV could be improved to 100 % by including the ratio of CD8^+^:PD-L1^+^ expressing cells.

**Conclusion:**

This is the first study to apply 7-color multispectral immunohistochemistry to analyze the immune environment of tumors from patients with melanoma. Assessment of the data using unsupervised hierarchical clustering identified tumors from which we were unable to generate TIL. If substantiated, this immune profile could be applied to select patients for TIL generation. Additionally, this biomarker profile may also indicate a pre-existing immune response, and serve as a predictive biomarker of patients who will respond to checkpoint blockade. We postulate that expanding the spectrum of inhibitory cells and molecules assessed using this technique could guide combination immunotherapy treatments and improve response rates.

**Electronic supplementary material:**

The online version of this article (doi:10.1186/s40425-015-0091-z) contains supplementary material, which is available to authorized users.

## Background

ACT with autologous TIL has shown great promise against metastatic melanoma, with response rates of up to 50–70 % and complete responses of up to 20 % [1–3]. In patients with melanoma, 95 % of the complete responders demonstrated a durable response for at least 5 years [1]. While the anti-tumor effect of ACT appears to be primarily mediated by CD8^+^ effector T cells [3–5], CD4^+^ T cells can also mediate tumor regression and may play a critical role in maintaining long-term immunity and cure of patients [6, 7]. T cells used for ACT are expanded from patients’ autologous tumors by *in vitro* culture with high-dose interleukin 2. Cultured TIL that recognize autologous tumor and secrete γ-interferon are considered autologous tumor-reactive. These cells are then cultured using a rapid expansion protocol (REP) and adoptively transferred into patients [5, 8–14]. A major limitation of adoptive T cell therapy is the inability to generate or expand tumor-reactive lymphocytes from many tumors. Autologous tumor-reactive T cells can be produced from 50 to 75 % of melanoma specimens, but success rates are much lower for other cancers (0–20 % for renal, breast and colon cancers) [15]. Identifying the reasons for failure of TIL isolation and expansion is important if we are to make ACT available to more patients with melanoma and other tumor types. Additionally, recent reports suggest that the response to checkpoint blockade agents such as anti-PD-1 and anti-PD-L1 is limited to patients with pre-existing immune responses [16, 17]. Since the isolation of autologous tumor-reactive TIL is potentially the best indicator that a T cell response against a patient’s tumor cells exists, we hypothesize that a pretreatment immunohistochemical assessment that can predict the ability to generate autologous tumor-reactive T cells may also serve as a biomarker to predict response to checkpoint blockade or other immunotherapies.

Quantitative immunohistochemistry has been useful for predicting response rates, treatment selection and determining prognosis in many types of cancer [17, 18]. This is especially notable in colon cancer, where the type and amount of tumor-infiltrating lymphocytes is highly predictive of prognosis [19, 20]. Similar reports have been made in melanoma, in which patients with high CD8^+^ T cells are associated with better prognosis [21–23]. Recently, multiplex immunohistochemistry (IHC) has emerged as an important tool for the analysis of the tumor microenvironment. Compared to traditional single color IHC methods, multiplex IHC methods are more efficient and contain richer information sets for both diagnostic and mechanistic studies [24, 25]. We utilized a multispectral quantitative fluorescent immunohistochemistry method, which allows simultaneous detection of 7 markers, to explore potential suppressive mechanisms in the tumor microenvironment that may prevent the generation of autologous tumor-reactive TIL. The results not only demonstrate for the first time the feasibility of this method in analyzing clinical tumor specimens, but also provide insight into possible reasons for the failure to isolate or expand autologous tumor-reactive TIL from patients with melanoma.

## Results

### Patients

Between 2001 and 2009, melanoma samples from 39 patients with melanoma enrolled in a cancer immunotherapy research study approved by the Providence Portland Medical Center’s (PPMC) Institutional Review Board, were used to generate TIL. Signed informed consent was obtained from all patients. Tumor specimen from 3 patients were excluded from our study, 2 of them are contaminated due to the site from which they were resected, and 1 was not able to be tested for tumor-reactivity because autologous tumor was unavailable. Surgical specimens for immunohistochemistry studies were obtained from the PPMC Pathology Department. Formalin-fixed-paraffin-embedded (FFPE) specimens and frozen section controls frozen section controls (FSC) samples from the same date were retrieved from Pathology for a subset of the patients whose tumors were resected at PPMC. FSC samples were prepared by thawing the frozen samples in OCT medium, fixing in 10 % formalin, and subsequently processed and embedded at PPMC pathology. In some cases, these reprocessed cryopreserved tumors were not useful for multispectral imaging and were excluded from our multispectral studies. Patient demographics are shown in Table 1. The median age was 56, with a range between 23 and 79; 65 % of the patients were male.

**Table 1:** Patient characteristics

Mel number	Sex	Successful expansion (N = 29)	Tumor-reactive (N = 21)	FFPE (N = 17)	Site	Time to growth (days)
Mel-119	M	No			Right Groin	
Mel-120	F	No				
Mel-131	M	Yes	Yes		Right Back	29
Mel-133	M	Yes	Yes	FFPE	Liver	30
Mel-134	M	Yes	Yes			38
Mel-135	F	No		FFPE	Right supraclavicular mass	
Mel-140	F	Yes	No			37
Mel-144	M	No		FFPE	Intraperitoneal mass	
Mel-145	M	Yes	Yes			27
Mel-150	F	No			Right back mass	
Mel-160	F	Yes	Yes	FFPE	Right Thigh	26
Mel-163 A	F	Yes	Yes	FFPE	Illiac LN	55
Mel-173-C	5 F	Yes	Yes	FSC		40
Mel-176	M	Yes	Yes	FFPE	lower left lobe	34
Mel-177A	F	Yes	Yes	FSC	Right Leg mass	25
Mel-179	F	Yes	No	FSC	Right axillary mass	56
Mel-180A	M	Yes	Yes		Axillary tumor mass	40
Mel-181	F	Yes	Yes			12
Mel-182	M	No		FFPE	Left pelvic mass	
Mel-185	M	Yes	Yes	FSC	Superior medistinal mass	33
Mel-186	M	No		FSC	Right lower lobe mass	
Mel-187	M	Yes	No		Paracenthesis fluid	67
Mel-188	M	Yes	No	FFPE	Right Axillary LN	41
Mel-189	M	No			Left Chest wall	
Mel-189A	M	Yes	Yes			32
Mel-190	M	No		FFPE	Small Bowl	
Mel-191	M	Yes	No		LN	28
Mel-192	F	No			Thigh	
Mel-193	F	No			Left Lung Upper Lobe	
Mel-199	M	Yes	Yes	FSC		48
Mel-200	M	Yes	Yes	FSC	Left lower tumor	37
Mel-201	M	Yes	Yes			26
Mel-206	M	Yes	Yes			56
Mel-207	M	Yes	Yes			35
Mel-208	M	Yes	Yes		Left lower lobe LN	33
Mel-209	M	Yes	Yes	FFPE	Liver mass	29
Mel-211	F	Yes	Yes		Liver mass	26

Tumor specificity is determined by IFN-γ release when stimulated with the autologous tumor cell line or tumor digest (defined as IFN-γ release greater than 100 pg/ml and double the background (T cells alone). FFPE or FSC (frozen section control) samples are available where indicated. The letters (A, B, C) after Mel-# designate tumors from different sites

### Comparison of methods to isolate and culture tumor-infiltrating lymphocytes

**Fig. 1:**
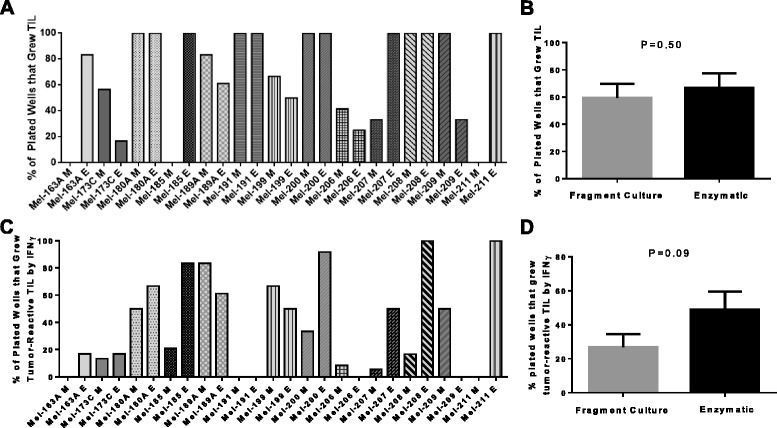
Efficiency of TIL Generation using tumor fragment and enzymatic digest. **a-b** Percentage of plated wells that grew TILs for (**a**) each patient and (**b**) average for all 13 patients. **c-d** Percentage of plated wells that grew tumor-reactive TILs for (**c**) each patient and (**d**) average of all 13 patients. Statistics are established using paired parametric analysis. M: tumor fragments; E: Enzymatic digest

The recent regulatory concerns regarding the source of enzymes used for enzymatic digestion of tumors may have led to an increased reliance on fragment cultures of tumor samples at cancer centers investigating TIL therapy. Fragment culture methods are faster and more cost effective and studies have reviewed the efficiency of fragment culture and enzymatic digest on different tumors [13]. However we are unaware of any head-to-head comparison of TIL generation from fragments and enzymatic digests of the same tumor. In this report, we compared the efficiency of fragment culture and enzymatic digestion in the generation of autologous tumor-reactive TIL cultures in 13 patients. A summary of results from this comparison is shown in Fig. 1. To generate TIL a minimum of 12 wells of a 24 well culture plate were initiated; 6 wells contained enzymatically digested tumor cell suspensions and 6 wells contained tumor fragments. Culture efficiency was evaluated in two ways. First, we determined the percentage of wells where it was possible to grow cultures of lymphocytes. Second, we evaluated whether the lymphocytes that grew out exhibited autologous tumor-reactive function, as defined by >100 pg/mL IFNγ release when stimulated with autologous tumor and at least two times the background of T cells cultured alone. While the ability to expand lymphocytes from tumors appeared equivalent between fragment culture and enzymatic digest isolation methods, there was a trend towards superiority for growth of autologous tumor-reactive lymphocytes with enzymatic digestion (P = 0.09). Importantly, we failed to generate autologous tumor-reactive TIL from 3 patients (23 %) using tumor fragment culture, and from 3 patients (23 %) when enzymatic digestion was used (Fig. 1c). If both methods were used to generate TIL, the success rate of generating an autologous tumor-reactive TIL culture improved to 92 % (12/13). While the numbers are small, this suggests that including both fragment culture and enzymatic digestion may enhance the success of tumor-reactive TIL isolation.

### Predicting the ability to generate autologous tumor-reactive TIL

**Fig. 2:**
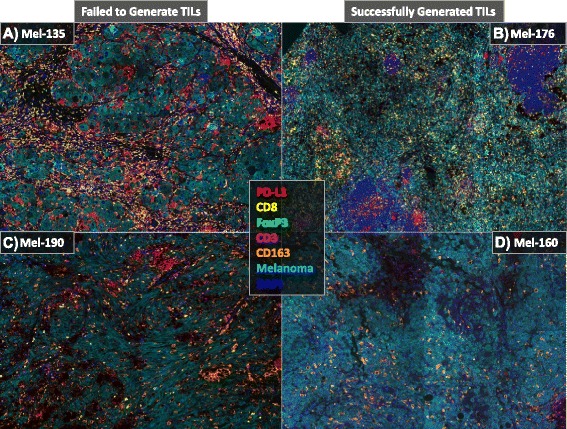
Sample 7-plex Images. Patients with high (Top) and low (Bottom) immune infiltrate from whom TIL did not (Left) or did (Right) grow. Images are 9 (Top) or 4 (Bottom) 200x fields stitched together for the specified melanoma specimens (**a-d**)

**Fig. 3:**
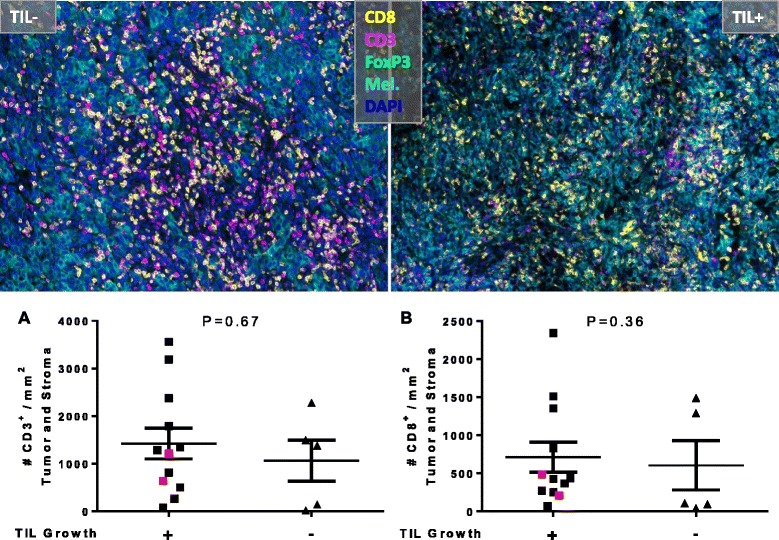
CD3 and CD8 are insufficient in predicting ability to generate TILs. Top: Pseudocolor H&E image of two example patients with similar CD3+ and CD8+ T cell infiltrate. Bottom: **a-b**) Quantification of the total percent of CD3+ and CD8+ T cells. Red colored dots indicated tumor sample that grew TIL but did not react to autologous tumor

We were able to culture autologous tumor-reactive TIL from 21/36 (58 %) of patients. This number is similar but a bit lower than the percentage of autologous tumor-reactive T cells generated from a large ACT clinical trial for treatment of melanoma (57/82, 70 %) [26]. The schematic for our culturing conditions is shown in Additional file 1: Figure S1. The patients from whose tumor we could not culture TIL were retested using cryopreserved tumor digest and the failure to grow TIL was confirmed. The first question we asked about the tumors from which TIL could not be grown was whether they were infiltrated by low numbers of CD8^+^ T cells. To address this question, we were able to retrieve 11 FFPE and 6 FSC blocks from our 36-patient cohort. Two blocks (Mel-179, Mel-188) represent cases which TIL could be successfully cultured but were not tumor-reactive; we included these in our study and they are highlighted in the figures for distinction. H&E sections from selected patients were blindly reviewed by a board-certified hematopathologist (C.B.B.) at our institute. Lymphocytic infiltrate within the tumor was classified as low (1+), intermediate (2+), or high (3+). No correlation was noticed between extent of lymphocytic infiltrate and the ability to generate TIL from the tumors (Additional file 1: Figure S2). From this we hypothesized that the tumor microenvironment in some patients is immunosuppressive such that the ability to culture TIL is limited. To address this question and further confirm our initial finding, we utilized a multispectral quantitative immunohistochemistry platform (Vectra, PerkinElmer, Hopkinton, MA) to more quantitatively assess the immune infiltrate of the tumors. Four representative images are shown in Fig. 2; there were two distinct patterns of immune infiltrate observed in our cohort: the patients with extensive immune infiltrates (Fig. 2a, b) and those with limited immune infiltrate (Fig. 2c, d). Supporting our initial observation with in Additional file 1: Figure S2, the extent of immune infiltrate alone was insufficient in predicting the ability to generate TIL (Fig. 3). We next examined specific immune markers for their ability to predict generation of TIL. We found that enumeration of the CD3^+^ and CD8^+^ T cell infiltrates alone is insufficient to predict successful generation of TIL. Representative images of tumors from two patients are shown in Fig. 3; although both tumors have high CD3^+^ (magenta) and CD8^+^ (yellow) T cell infiltrate, we were unable to culture TIL from the tumor on the left, while we were able to generate TIL from the tumor on the right.

**Fig. 4:**
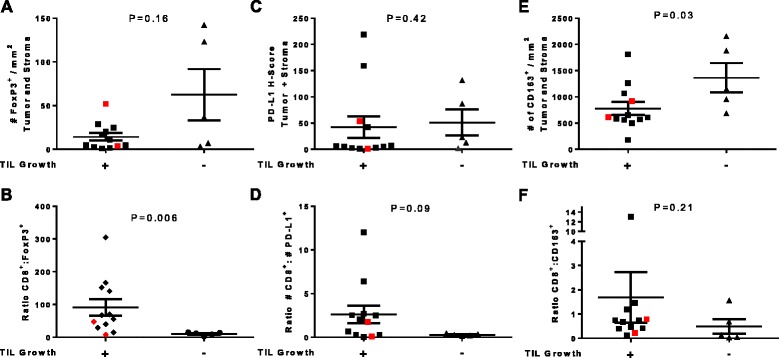
CD8 to FoxP3 ratio is predictive of ability to culture autologous TILs. **a-b** FoxP3 alone and CD8:FoxP3 ratio. **c-d**) PD-L1 alone and CD8:PD-L1 ratio. **e-f**) CD163 alone and CD8:CD163 ratio. Statistics are done with unpaired nonparametric *T* test (Mann–Whitney Ranked Comparison). Significance is established at P < 0.05. Red colored dots indicated tumor sample that grew TIL but did not react to autologous tumor

**Fig. 5:**
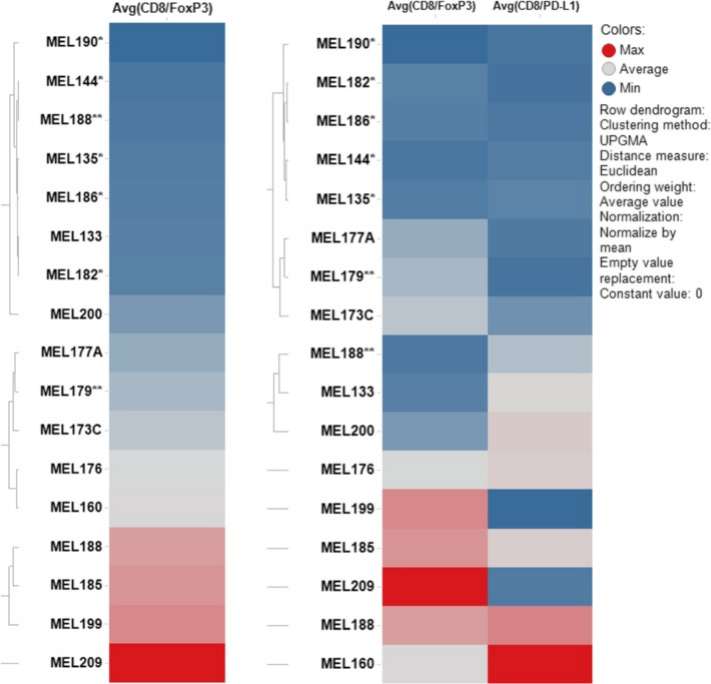
Unsupervised hierarchical clustering of CD8+:FoxP3+ and CD8+:PD-L1+ ratios. The color is a continuing spectrum with dark red indicating max expression and dark blue indicating minimum expression. *Indicates patients from whom we failed to culture TIL. **Indicates tumor sample that grew TIL but did not react to autologous tumor

Considering the failure to generate TIL may be a consequence of an immune-suppressive environment, we evaluated tumors for the number of CD3^+^FoxP3^+^ regulatory T cells, and CD163^+^ alternatively activated (M2) macrophages; and also quantified the number of cells expressing PD-L1. As PD-L1 can also be expressed on melanoma cells (Additional file 1: Figure S4A), we used a cocktail consisting of HMB45, Mart-1 and Tyrosinase to identify tumor cells. The cocktail was positive in 70 % of the patients in our cohort. Since PD-L1 can also be expressed on CD163^+^ macrophages, and CD3^+^CD8^+^ T cells (Additional file 1: Figure S4B-C), we included their expression in our evaluation as well. We analyzed the percent of CD3^+^FoxP3^+^ regulatory T cells, percent of CD163^+^ macrophages, and PD-L1 expression using H-Score, a value based both on the intensity of the PD-L1 expression on a 0-3+ scale, and the number of cells that are positive for PD-L1. We did not find any significant correlation between the amount of CD3^+^FoxP3^+^ regulatory T cells and the ability to generate TIL. However, when we evaluated the relative proportion of CD8^+^ and CD3^+^FoxP3^+^ regulatory T cells, we found that the ratio was highly significant (P = 0.006, PPV = 91 %) in predicting TIL culture success (Fig. 4b). This suggests that the percent of regulatory T cells present in the tumor may be a determining factor in limiting the proliferation of T cells *in vitro.* PD-L1 expression by itself did not correlate with the ability to culture TIL, however, when we took the ratio of CD8^+^ T cells to PD-L1^+^ cells, there was an increased trend(P = 0.09) (Fig. 4c-d), suggesting a potential contributory immunosuppressive roles for PD-L1 in preventing the generation of TIL *in vitro*. We also found an inverse correlation between the number of CD163^+^ macrophages and the ability to culture TIL (P = 0.03). This correlation, was however not further improved when the number of CD8^+^ T cells was taken into account (Fig. 4e-f). We then constructed a heat map using all available data sets (Additional file 1: Figure S7) and performed an unsupervised hierarchical analysis on all samples (Fig. 5). We found that the CD8^+^ to PD-L1^+^ ratio was able to further enhance the NPV of CD8^+^ to FoxP3^+^ ratio from 86 to 100 %. Neither positive nor negative predictive values were further improved by the amount of CD163^+^ infiltrate.

## Discussion

Adoptive T cell therapy using autologous T cells has been shown to be an effective way to treat some patients with melanoma. The infrastructure and laboratory support required is quite substantial and has limited TIL therapy to a small number of academic sites. A further limitation is that the success rate for production of tumor-reactive TIL in melanoma has been between 50 and 70 %. Thus this therapy is currently limited to a subset of patients with melanoma from whom tumor-reactive TIL can be isolated and expanded. Methods that improve the success rate of culturing tumor-reactive TIL might increase the availability of ACT to more patients with melanoma, and perhaps other malignancies. The two standard methods of processing tumor for TIL generation are culture of tumor fragments and enzymatic digestion of tumor fragments with culture of the isolated tumor cell suspension. We have compared both methods for their efficiency in generating tumor-reactive TIL using fragment culture and enzymatic digestion and found a trend favoring increased efficiency using enzymatic digest. Importantly, we found that regardless of the method there was a failure to produce clinically useful T cells for adoptive immunotherapy in one of every three patients. Our data suggest that employing both methods increased the chances for success and could maximize the number of patients from whom TIL can be grown. In addition, time in culture seemed to play an important role, as previously reported [27]. We found an inverse correlation between the autologous tumor-reactivity of TIL and the amount time they had spent in culture (Additional file 1: Figure S4).

The second main aim of our study was to analyze the tumor microenvironment for factors that may limit the generation of tumor-reactive TIL. We utilized a novel multispectral immunohistochemistry method employing the PerkinElmer Vectra platform to examine the microenvironment of melanoma. Part of our aim was to test the feasibility of this method to analyze multiple markers on a single 4-micron section of a FFPE tumor sample. Overall, we found that the method was reproducible and permitted the simultaneous detection of up to 7 markers (Fig. 2, Additional file 1: Figure S8). We have compared two different methods for image analysis and found no significant difference between using the percentage of immune infiltrate or the number of immune infiltrating cells per mm^2^ (Additional file 1: Figure S3A). We found no significant difference in the results of the immune infiltrate analysis whether we used the PerkinElmer inform software or the Definiens Tissue Studio (Additional file 1: Figure S3B). The method works best with FFPE samples, which we could retrieve from 11 patients. For frozen sections that are subsequently fixed with formalin, certain markers such as CD8 can demonstrate an artifactual punctate pattern of positivity (Additional file 1: Figure S6F, black arrows), which increased the difficulty of the digital morphometric analysis. We also found that FoxP3 staining in frozen sections had a higher background and can lead to over-estimation of the number of FoxP3^+^ cells due to artifacts (Additional file 1: Figure S6I, black arrows). Since the artifacts are not CD3^+^, this problem was circumvented in our study by using both CD3 and FoxP3 to determine the number of regulatory T cells and including only the cells that were positive for both CD3 and FoxP3 (Additional file 1: Figure S6I, red arrows). Detection of CD163, CD3 and PD-L1 did not seem to be affected to the same degree by artifactual changes induced by freezing (Additional file 1: Figure S6B, C, E). Detection of melanoma cells by the melanoma cocktail was only positive in 70 % of specimens. An alternative approach to improve detection may be the use of an antibody against SOX10, which has shown superior sensitivity and specificity over the melanoma cocktail [28].

From our multispectral analysis, we found that the presence of CD8^+^ T cells alone was insufficient in predicting the success of generating a TIL culture, while the ratio of CD8^+^ T cells to CD3^+^FoxP3^+^ regulatory T cells was a significant predictor (P = 0.006). This is consistent with previous reports in preclinical models showing that depletion of FoxP3^+^ regulatory T cells, or inhibiting regulatory T cell function through OX40 or GITR activation, can lead to an increased CD8: Foxp3^+^ T cell ratios that correlated with improved CD8^+^ T cell function and therapeutic efficacy [29–31]. In other studies, regulatory T cells isolated from tumor were shown to directly inhibit T cell function in non-small cell lung cancer [32], and may negatively influence the response rate of patients receiving ACT [33, 34]. We also found that the number of CD163^+^ macrophages inversely correlated with the ability to culture TIL (Fig. 4e); consistent with reports suggesting depletion of macrophages through inhibition of CSF1R signaling inhibition can enhance CD8^+^ T cell function in preclinical models [35–37]. This has led to a phase I clinical trial evaluating anti-CSF-1R administration, which identified partial clinical responses in 5/7 patients with diffuse-type giant cell tumor [38]. We evaluated the effect of arginase I and iNOS inhibition during the generation of TIL cultures but found no significant increase in recovery success (Puri et al. manuscript in preparation). Recently, other have shown that by using an agonist 4-1BB antibody during the initialing of culture increased the numbers of memory CD8^+^ TIL that were specific for autologous tumor and represents a promising approach to increase the number of patients eligible to receive adoptive immunotherapy with TIL [39].

While PD-L1 expression by itself is not an indicator of TIL culture success, the ratio of CD8^+^ T cells to PD-L1^+^ cells is trending towards a significant separation, suggesting this important negative feedback loop may limit the ability to grow tumor-reactive TIL *ex vivo.* We subsequently performed unsupervised clustering of the data, which allowed us to further analyze the large amount of information generated with multispectral imaging. From this analysis, we were able to incorporate 2 out of 3 suppressive markers from our panel and identify a cluster of patients whose tumor demonstrated both low CD8^+^:FoxP3^+^ and low CD8^+^:PD-L1^+^ ratios (Fig. 5); and we failed to generate TIL from these tumors. Our observations raised the question whether CD8^+^ T cell proliferation was suppressed during culture, or whether they were already anergic and unable to proliferate at the time they were placed in culture due to pre-existing suppressive mechanisms. Evidence from murine and human studies suggests that expression of PD-L1 and the presence FoxP3^+^ regulatory T cells can reduce the anti-tumor effect of ACT [33, 34, 40, 41]. These studies imply that regulatory T cell depletion and use of anti-PD-L1 antibody before culture may increase the success rate in growing tumor-reactive TIL, and potentially expand the use of ACT to greater number of patients with melanoma and potentially other malignancies.

Checkpoint blockade therapy is one of the most promising treatments for patients with solid tumors. Recent studies suggest that the response to agents such as anti-PD-1 and anti-PD-L1 is limited to patients with pre-existing immune response [16, 17]. We consider the ability to culture tumor-reactive lymphocytes from tumors of patients to be a very specific indicator of a pre-existing anti-cancer immune response. The density of CD8^+^ T cells has shown to be a powerful marker in predicting response to anti-PD-1 therapy in a small cohort of patients, correctly predicting 13/15 patients who are treated with anti-PD-1 therapy [16]. It is possible that including FoxP3^+^ regulatory T cells may further increase the prediction for anti-PD-1 response. We plan to apply our multispectral immunohistochemical analysis to patients receiving T-cell checkpoint antibodies.

## Conclusion

In summary, this is the first study to apply 7-color multispectral immunohistochemistry to analyze the immune environment of tumors from patients with melanoma. Enumeration of staining with objective assessment software and analysis of the data using unsupervised hierarchical clustering identified tumors where we were unable to generate TIL. These results could be evaluated with a prospective study to determine if this immune profile could select patients for successful TIL generation. Additionally, since this biomarker profile appears to identify presence of tumor-reactive T cells, it may represent a predictive biomarker of patients who will respond to checkpoint blockade. While application of this methodology is at an early stage, we consider that its greatest promise will be as a means to identify resistance mechanisms operational at the tumor site. In this era of combination immunotherapy, this information will be a useful guide to tailor specific therapies to patients with cancer.

## Methods

### TIL generation

Tumor procurement and processing: All tumors were resected as part of an IRB-approved protocol of the Providence Portland Medical Center and were numbered sequentially upon arrival in the Human Applications Laboratory (HAL) of the Earle A. Chiles Research Institute (EACRI). Appropriate consent were collected from patients from whom the tumors were collected for research use. At the beginning of these studies, all tumors were processed by triple enzyme digestion using a mixture of collagenase type IV (Cat# C-5138, SIGMA), hyaluronidase type-V (Cat# H-6254, SIGMA) and deoxyribonuclease-1 (Cat# D-5025, SIGMA). Subsequently, tumors were processed using GMP manufactured enzymes that included Liberase MTF C/T, a combination of collagenase and thermolysin (Cat# 05339880529, Roche), Hylenex (Baxter) or Amphadase (Amphaster), and DNase-1 recombinant grade-1 (Cat# 04536282001, Roche). The freshly minced tumor suspension and enzyme mixture was subsequently mixed at room temperature for 4 to 18 h, using a magnetic stir bar and stir plate with rotations set at the lowest speed that would keep the tumor fragments suspended. The resulting tumor digest was filtered through a 200 micron nylon membrane and washed twice with HBSS, counted and resuspended in Human AB culture medium. Human AB culture medium (CM) is comprised of RPMI 1640 (Lonza,Walkersville,MD), 25 mmol/L HEPES pH 7.2 (Lonza), L-Glutamine (200 mM) (Lonza), penicillin/streptomycin 10,000 units/ml (Lonza), gentamicin 50 mg/mL (Cambrex), 5.5 × 10 − 5 M β-mercaptoethanol (Life Technologies, Eugene,OR), supplemented with 10 % heat-inactivated human AB serum (Valley Biomedical, Inc, Winchester, VA; Cat #HP1022; Lot numbers K-61552, G-81460 and B-90211). These lots of human AB serum were screened by the Surgery Branch, NCI, NIH and shown to support generation of human TIL (Screening information supplied by Dr. Maria Parhkurst and Linda Parker).

For the head-to-head comparison of TIL generation from tumor fragments and enzymatic digests, the same tumor specimen was minced into 1–2 mm^2^ fragments and representative fragments were taken to set up fragment TIL cultures. The remainder of the minced tumor was processed for isolation of cells by enzymatic digestion.

Briefly, TIL generation was performed similarly to that outlined by Surgery Branch, NCI, NIH, tiltum protocol 9-6-05 (provided by DR. John Wunderlich). Typically, at least 6 wells of tumor fragments and 6 wells enzymatically digested tumor were plated for culture. For tumor fragment cultures, typically 4 to 10 fragments were plated into each well of a 24 well plate containing 2 mL human AB CM supplemented with 1000 cU/mL. Enzyme digested tumor suspensions were adjusted to 5.0 × 10^5^/mL in human AB CM supplemented with 1000 cU/ml IL-2 and 2mls were plated per well of a 24 well plate. The 24 well plates were placed in a humidified 37 °C incubator with 5 % CO2 and cultured until lymphocyte growth was evident. Each well of the plate was inspected on alternate days using a low-power inverted microscope to monitor the extrusion and proliferation of lymphocytes. Whether or not lymphocyte growth was visible, half of the medium was replaced in all wells no later than 1 week after culture initiation. Typically, about 1 to 2 weeks after culture initiation, a dense lymphocytic carpet would cover a portion of the plate surrounding each fragment. When any well became almost confluent, all the growing lymphocytes were mixed vigorously, split into two daughter wells and filled to 2 mL per well with CM plus 1000 cU/mL IL-2. Subsequently, the cultures were split to maintain a cell density of 0.5 to 1.0 × 10^6^ cells/mL, or half of the media was replaced at least twice weekly or (as needed). The age of TIL cultures used in these studies varied from 12 to 67 days. Each culture originating from each of the initial 6 wells plated for each condition were considered to be an independent TIL culture or “cloid” and maintained in a separate plate with separate pipettes used to maintain integrity of each cloid during expansion.

### Cytokine release assays (IFNγ Release)

Tumor specificity or reactivity assays were initiated the same day cells were harvested and frozen for future use. TIL activity and specificity were determined by analysis of IFNγ secretion following stimulation with autologous tumor cells. TIL (1 × 10^6^ cells/well) were plated in a 24-well plate with 2.5 × 10^5^ autologous stimulator tumor cells/well. Autologous tumor cells were either cryopreserved enzymatic tumor digests or autologous melanoma cell lines. Control wells contained either TIL alone or tumor cells alone. Supernatants were harvested after 18–20 h and IFNγ secretion was measured by enzymes linked-immunosorbent assay (ELISA) technique according to manufacturers guidelines (eBioscience) TIL. were considered autologous tumor-reactive if at least one of the TIL cloids released >100 pg/ml of IFNγ and this value was > twice the background values (IFNγ release from TIL alone).

### Immunohistochemistry

4 μm thick sections were prepared and stained using PerkinElmer Opal kit. Slides were scanned using the PerkinElmer Vectra and images were analyzed using the inForm software (PerkinElmer, Hopkinton, MA). The rabbit anti-human CD8 monoclonal antibody (SP239, M5394 Spring Biosciences) and anti-human CD163 monoclonal antibody (MRQ26, 760–4437, Ventana) were both kind gifts of Ms. Alisa Tubbs (Ventana, Tucson, AZ). For a detailed protocol see “Additional file 2”.

### Statistical analysis

Significance between groups are established using unpaired *T* test performed in Graphpad Prism. Unsupervised hierarchical analysis is performed using TIBCO Spotfire, UPGMA clustering method.

## Additional files

Additional file 1: Figure S1. Schematic representation of process for TIL initiation, expansion and testing. **Figure S2.** Lymphocytic immune infiltrate is insufficient to predict TIL culture success. A) Representative H&E stain from 4 patients, two are classified as 3+ (Top) and two are classified as 1+ (Bottom), TIL culture status is indicated above images. B) Compares average pathology scores of lymphocytic infiltrate between tumors that grew TILs and those that did not. **Figure S3.** Comparison of two methods to evaluate immune infiltrate. A) Comparison between % CD8 T cell infiltrate and # of CD8 positive cells/mm^2. B) Comparison between # of CD8 positive T cells/mm^2 determined using PerkinElmer and Definiens. The comparisons were done using the identical image. Statistics were determined using Prism. **Figure S4.** PD-L1 Localization. A) On tumor cells B) on CD8+ T cells and C) on CD163+ macrophages. **Figure S5.** Time of growth is predictive of tumor reactivity. Statistics were done using unpaired nonparametric *T* test. Significance was established at P < 0.05. **Figure S6.** Comparison between FFPE (Top) and frozen (Bottom) sections. A,F) CD8; B,G) CD3; C,H) CD163; D,I) FoxP3; E,J) PD-L1. **Figure S7.** Heatmap representation of parameters generated from multispectral imaging with dark red indicating maximal expression and dark blue indicating minimum expression. *indicates patients from whom we failed to culture TIL. **indicates tumor sample that grew TIL that were not reactive to autologous tumor. **Figure S8.** Multispectral image showing individual channels: Original is the raw image with all channels taken using the Vectra imaging software. The spectrum for each fluorophore is measured with control slides and subtracted from the original image to establish individual channels and the composite. **Figure S9.** Representative image from individual multispectral samples. TIL status is indicated with “+” or “-“. Enumerated immune infiltrates are indicated for each sample in the lower right corner. (PPTX 38563 kb) Additional file 2:
**Supplemental methods.** Detailed immunohistochemistry methods for multispectral analysis. (DOCX 20 kb)

## Abbreviations

ACT: Adoptive cellular therapy
CD: Cluster of differentiation
CM: Complete medium
FFPE: Formalin-fixed paraffin-embedded
FoxP3: Forkhead box P3 also known as scurfin
FSC: Frozen section controls
GITR: Tumor necrosis factor receptor superfamily member 18 (TNFRSF18) also known as activation-inducible TNFR family receptor (AITR)
HAL: Human applications laboratory
H&E: Hematoxylin and eosin
IHC: Immunohistochemistry
iNOS: Calcium-insensitive Nitric oxide synthases
IU: International unit
NPV: Negative-predictive value
OX40: Tumor necrosis factor receptor superfamily, member 4 (TNFRSF4), also known as CD134
PD-1: Programmed cell death protein 1, also known as CD279
PD-L1: Programmed death-ligand 1 also known as CD 274 or B7 homolog 1 (B7-H1)
PPMC: Providence Portland Medical Center
PPV: Positive-predictive value
REP: Rapid expansion protocol
TIL: Tumor-infiltrating lymphocytes
