# Supplementary material for: Multispectral imaging of formalin-fixed tissue predicts ability to generate tumor-infiltrating lymphocytes from melanoma
Source: J Immunother Cancer. 2015 Oct 20;3:47. doi: 10.1186/s40425-015-0091-z (PMC4617712; doi:10.1186/s40425-015-0091-z)
Supplement: Additional file 1: Figure S1. — Schematic representation of process for TIL initiation, expansion and testing. Figure S2. Lymphocytic immune infiltrate is insufficient to predict TIL culture success. A) Representative H&E stain from 4 patients, two are classified as 3+ (Top) and two are classified as 1+ (Bottom), TIL culture status is indicated above images. B) Compares average pathology scores of lymphocytic infiltrate between tumors that grew TILs and those that did not. Figure S3. Comparison of two methods to evaluate immune infiltrate. A) Comparison between % CD8 T cell infiltrate and # of CD8 positive cells/mm^2. B) Comparison between # of CD8 positive T cells/mm^2 determined using PerkinElmer and Definiens. The comparisons were done using the identical image. Statistics were determined using Prism. Figure S4. PD-L1 Localization. A) On tumor cells B) on CD8+ T cells and C) on CD163+ macrophages. Figure S5. Time of growth is predictive of tumor reactivity. Statistics were done using unpaired nonparametric T test. Significance was established at P < 0.05. Figure S6. Comparison between FFPE (Top) and frozen (Bottom) sections. A,F) CD8; B,G) CD3; C,H) CD163; D,I) FoxP3; E,J) PD-L1. Figure S7. Heatmap representation of parameters generated from multispectral imaging with dark red indicating maximal expression and dark blue indicating minimum expression. *indicates patients from whom we failed to culture TIL. **indicates tumor sample that grew TIL that were not reactive to autologous tumor. Figure S8. Multispectral image showing individual channels: Original is the raw image with all channels taken using the Vectra imaging software. The spectrum for each fluorophore is measured with control slides and subtracted from the original image to establish individual channels and the composite. Figure S9. Representative image from individual multispectral samples. TIL status is indicated with “+” or “-“. Enumerated immune infiltrates are indicated for each sample in the lower right corner. (PPTX 38563 kb) [file 40425_2015_91_MOESM1_ESM.pptx]

## Slide 1
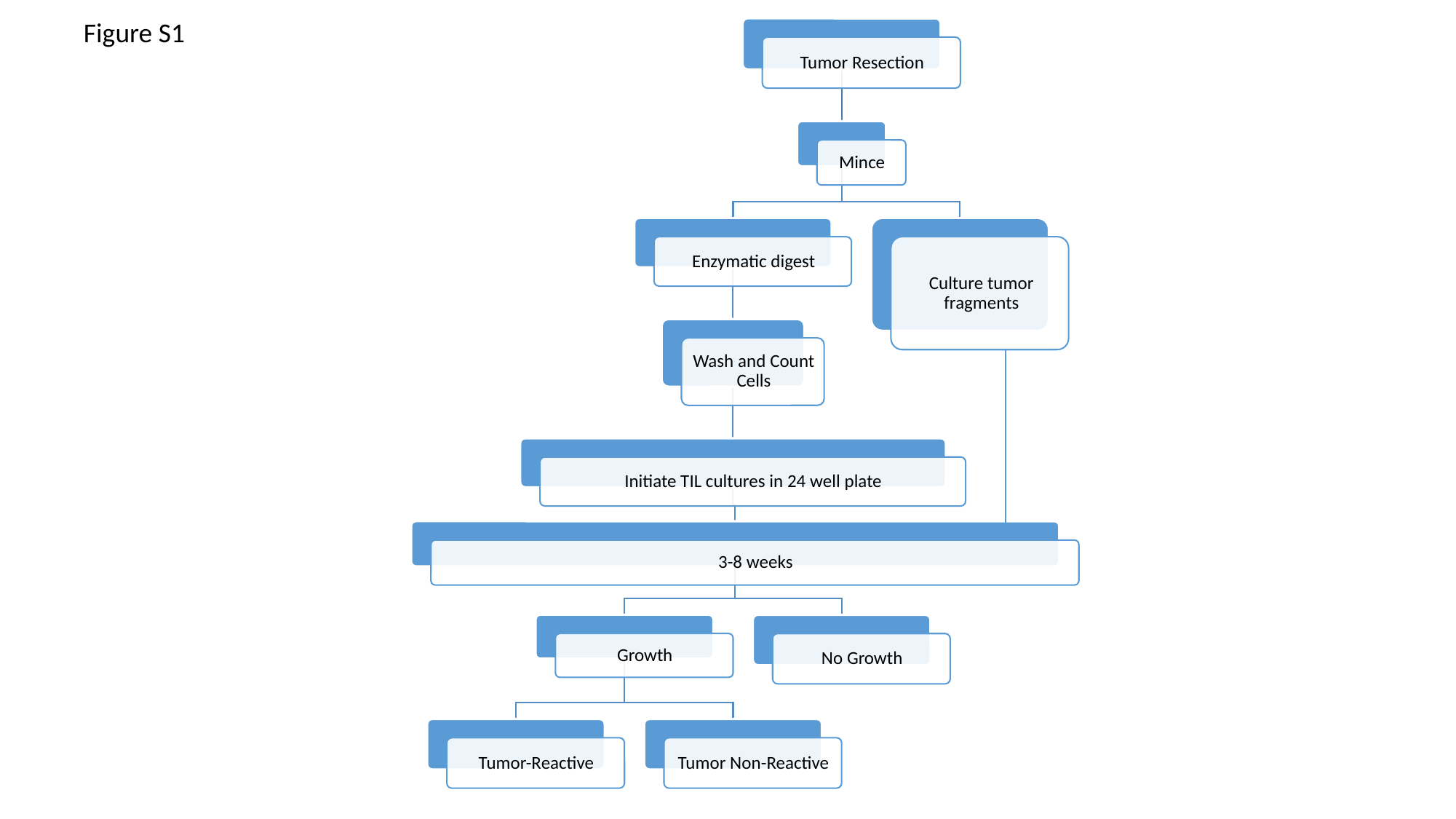

Figure S1

## Slide 2
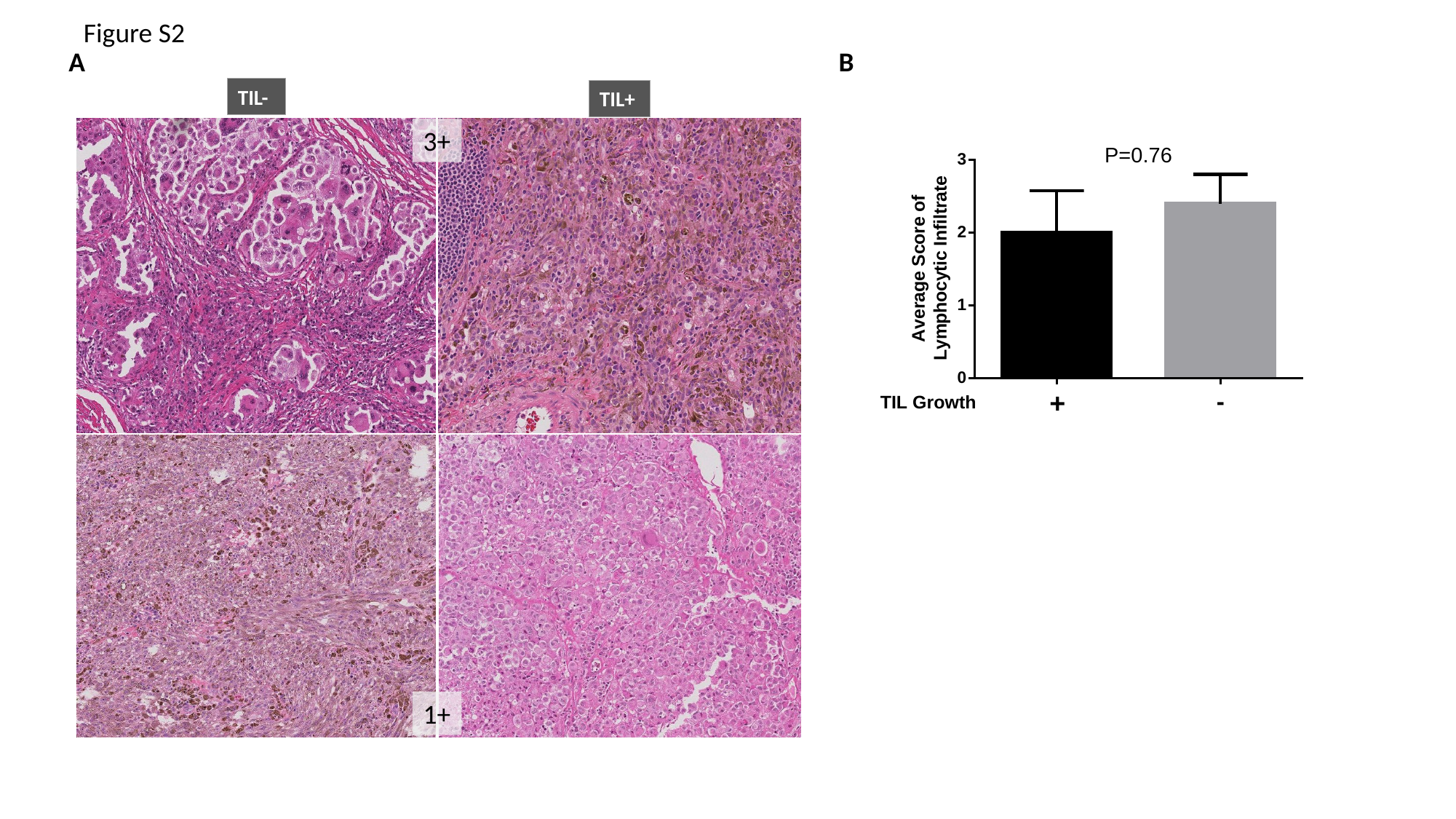

Figure S2
A
B
TIL-
TIL+
3+
1+

## Slide 3
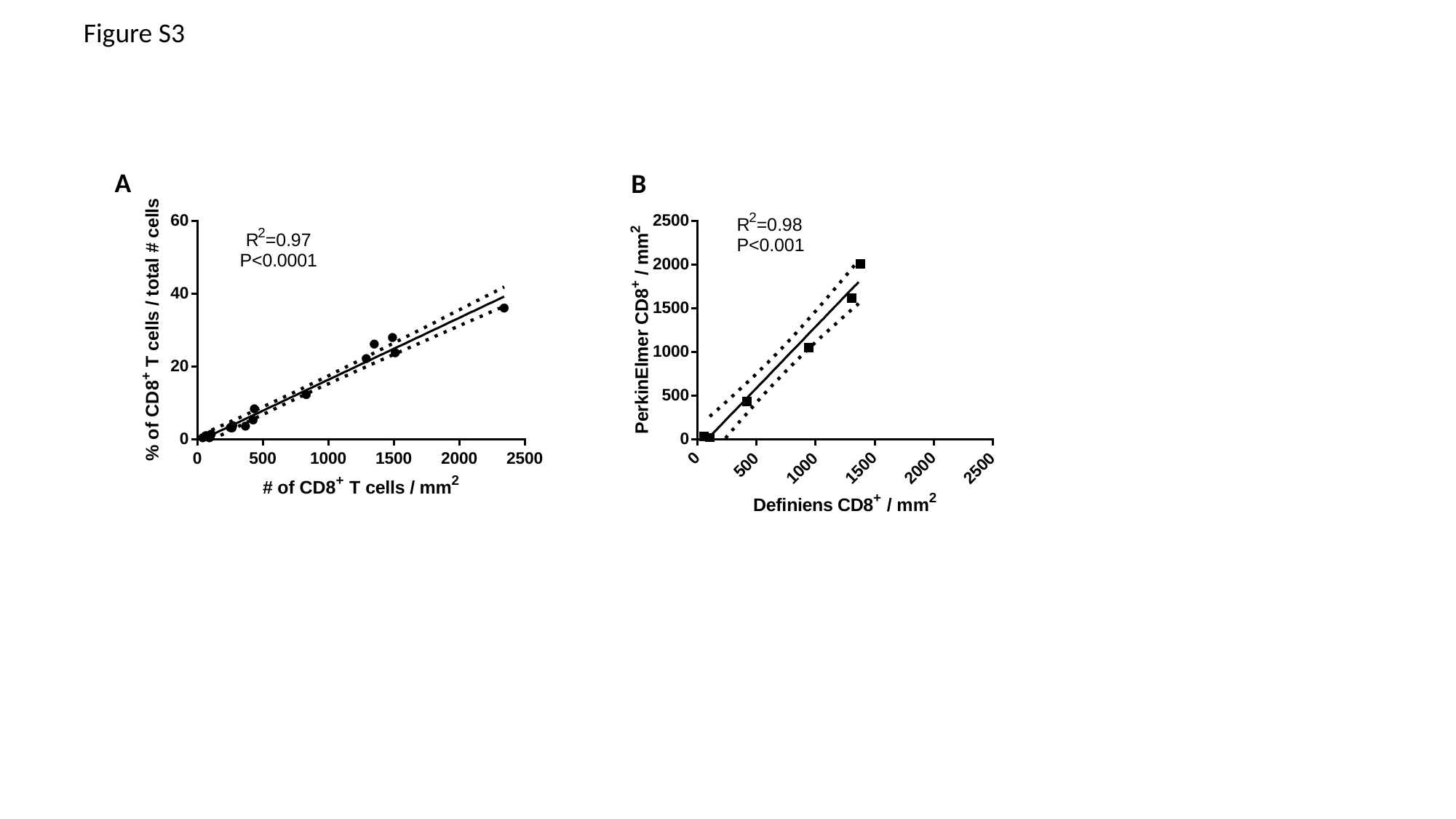

Figure S3
A
B

## Slide 4
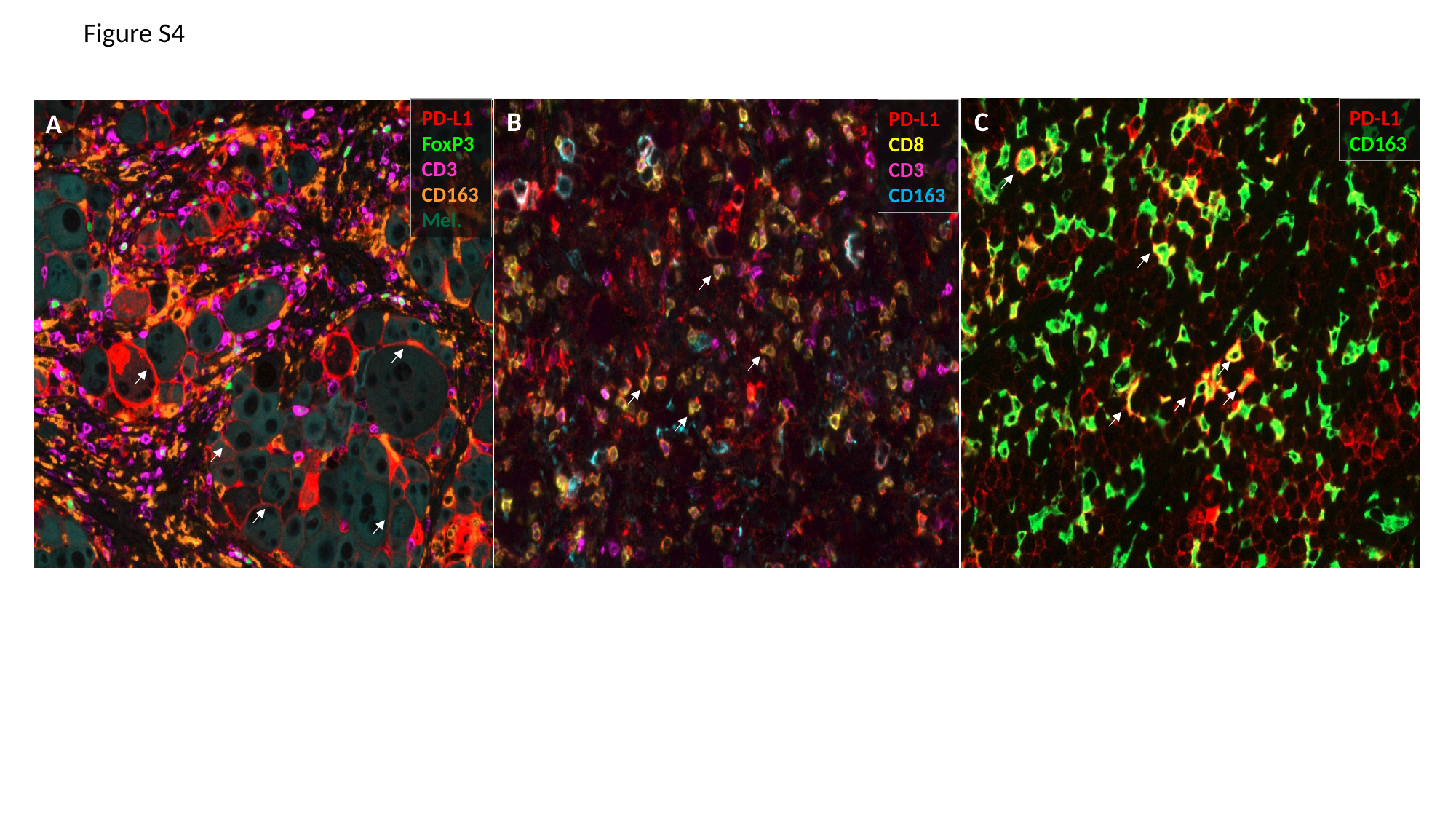

Figure S4
PD-L1
FoxP3
CD3
CD163
Mel.
B
C
PD-L1
CD163
PD-L1
CD8
CD3
CD163
A

## Slide 5
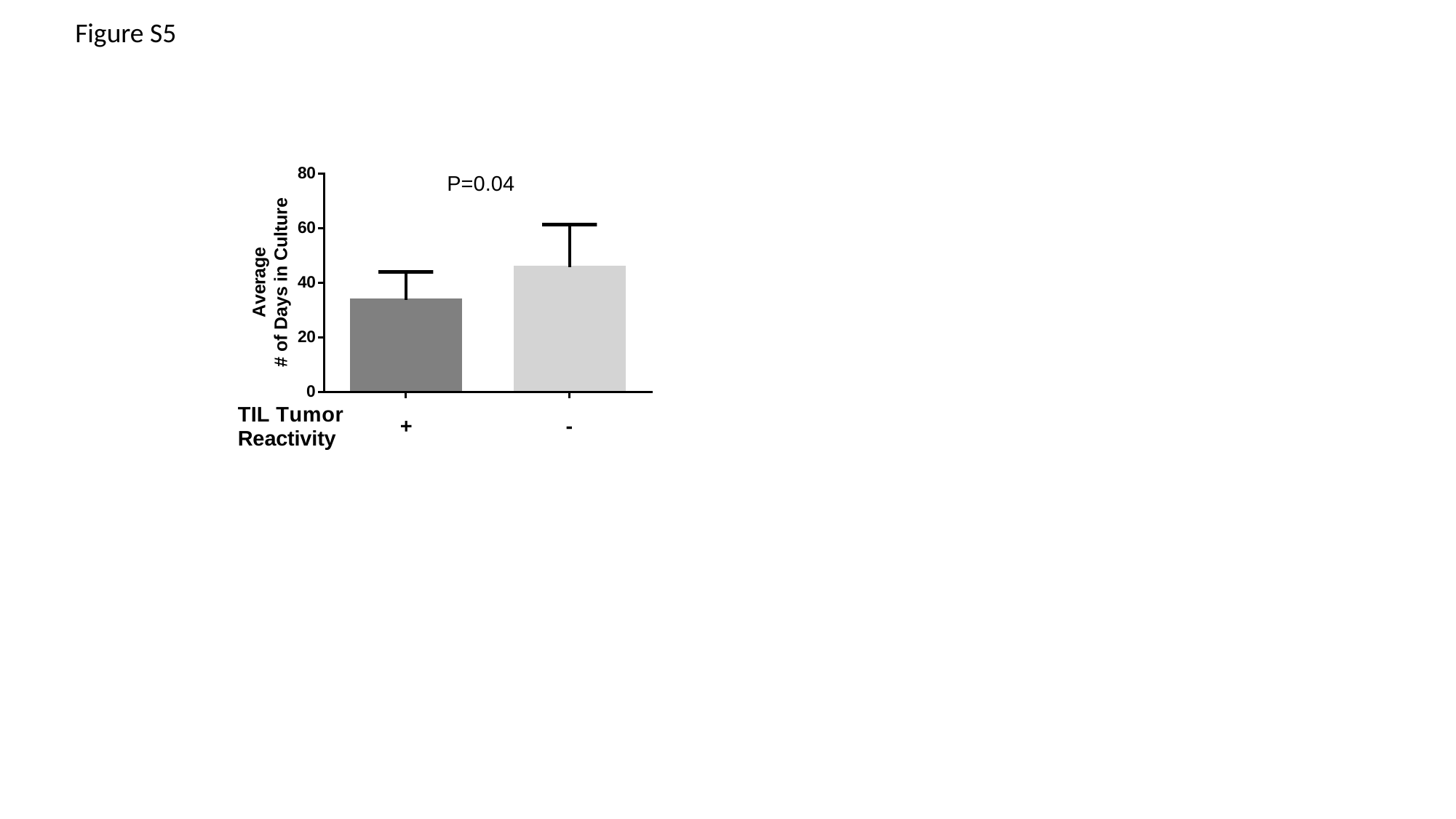

Figure S5

## Slide 6
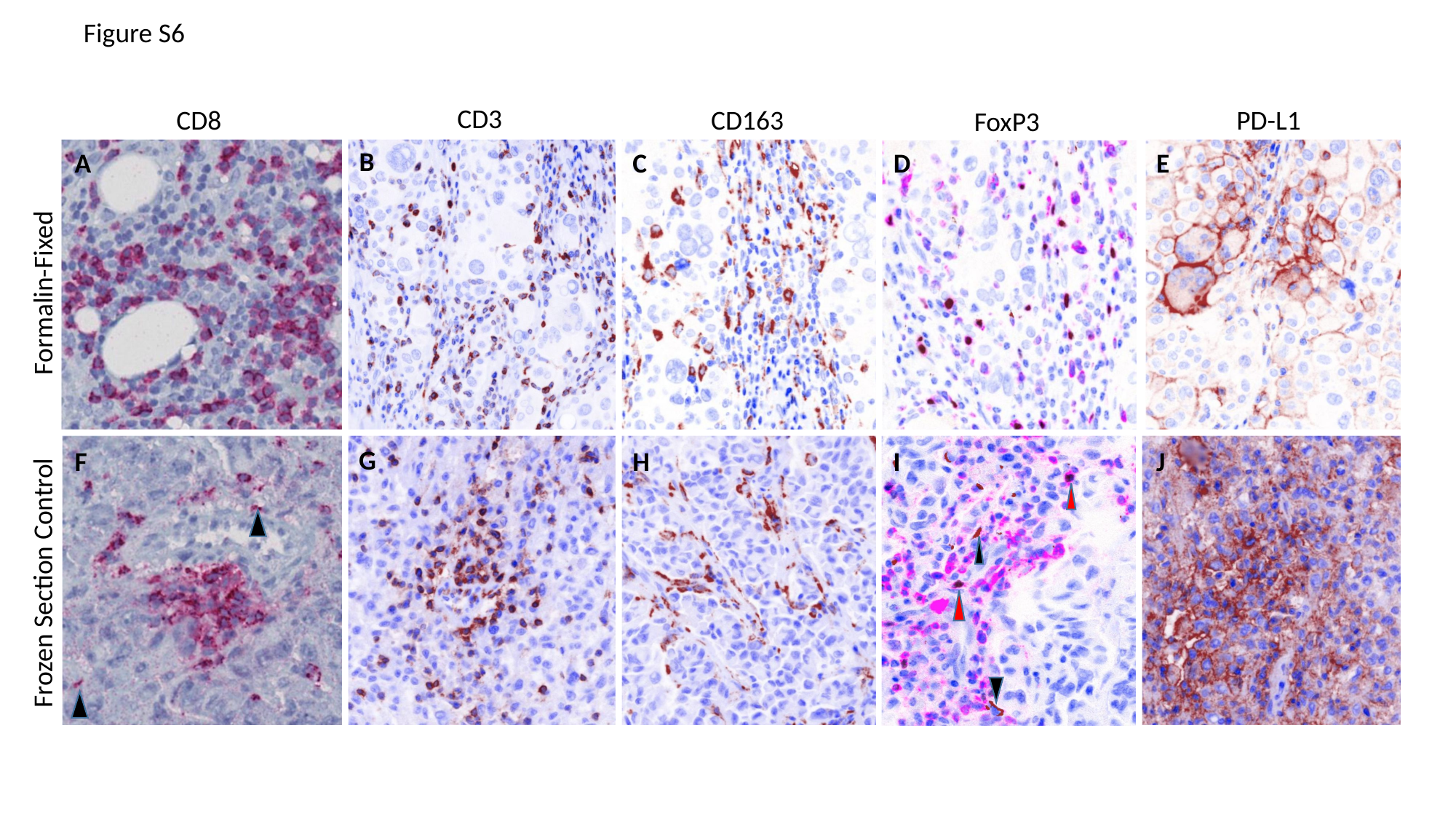

Figure S6
CD3
PD-L1
CD8
CD163
FoxP3
B
A
C
D
E
Formalin-Fixed
G
F
H
I
J
Frozen Section Control

## Slide 7
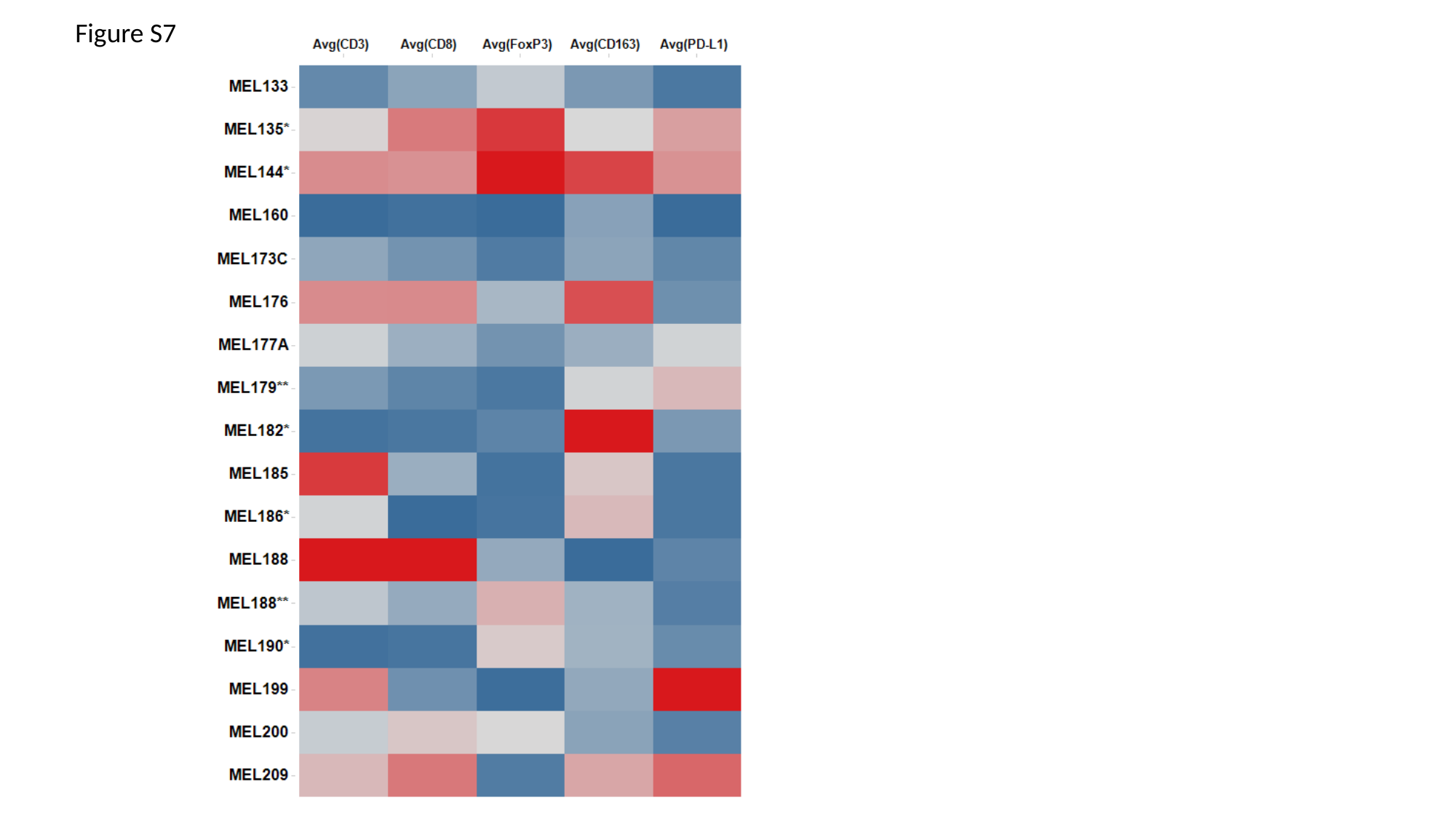

Figure S7

## Slide 8
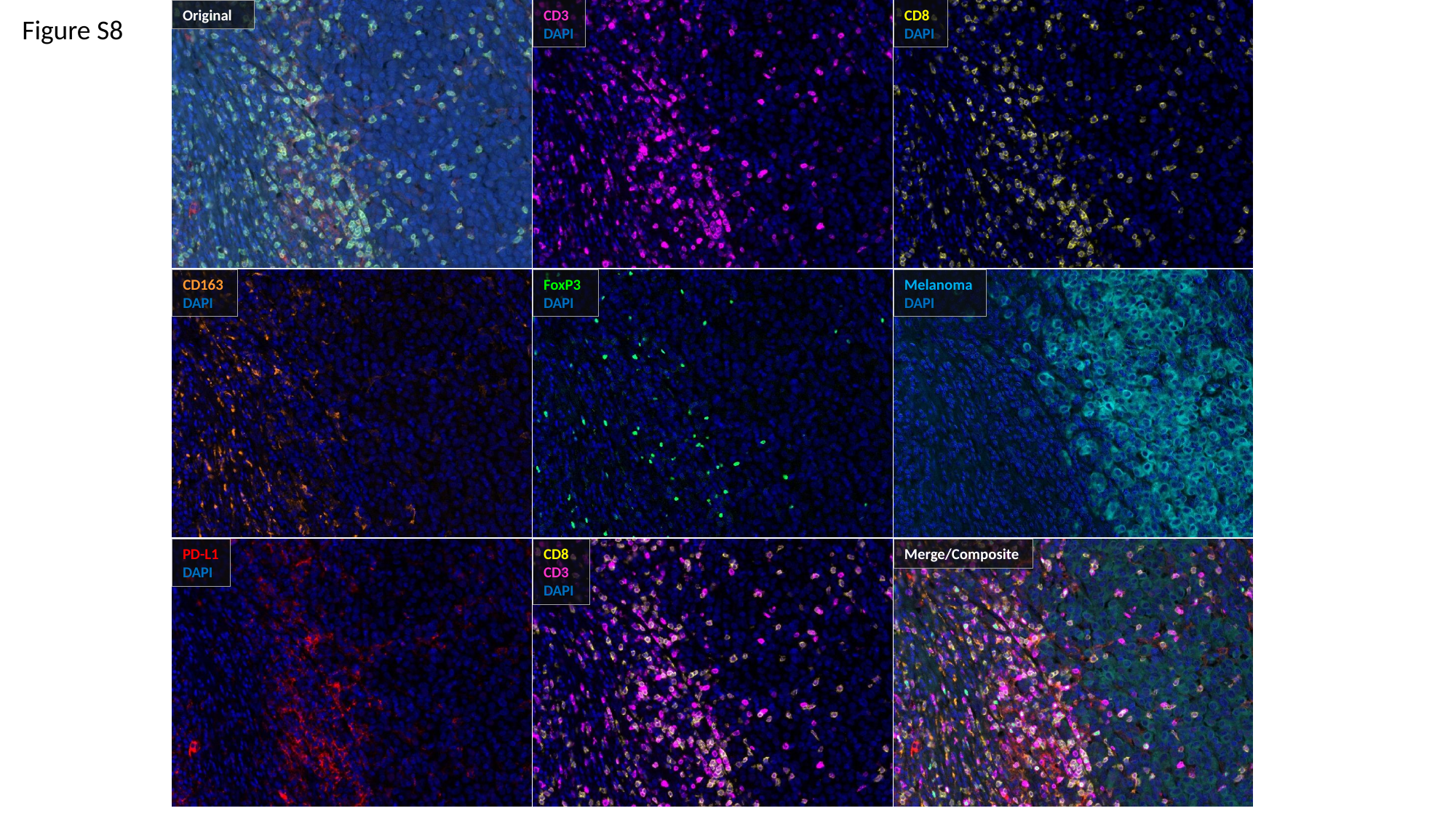

Original
CD3
DAPI
CD8
DAPI
CD163
DAPI
FoxP3
DAPI
Melanoma
DAPI
PD-L1
DAPI
CD8
CD3
DAPI
Merge/Composite
Figure S8

## Slide 9
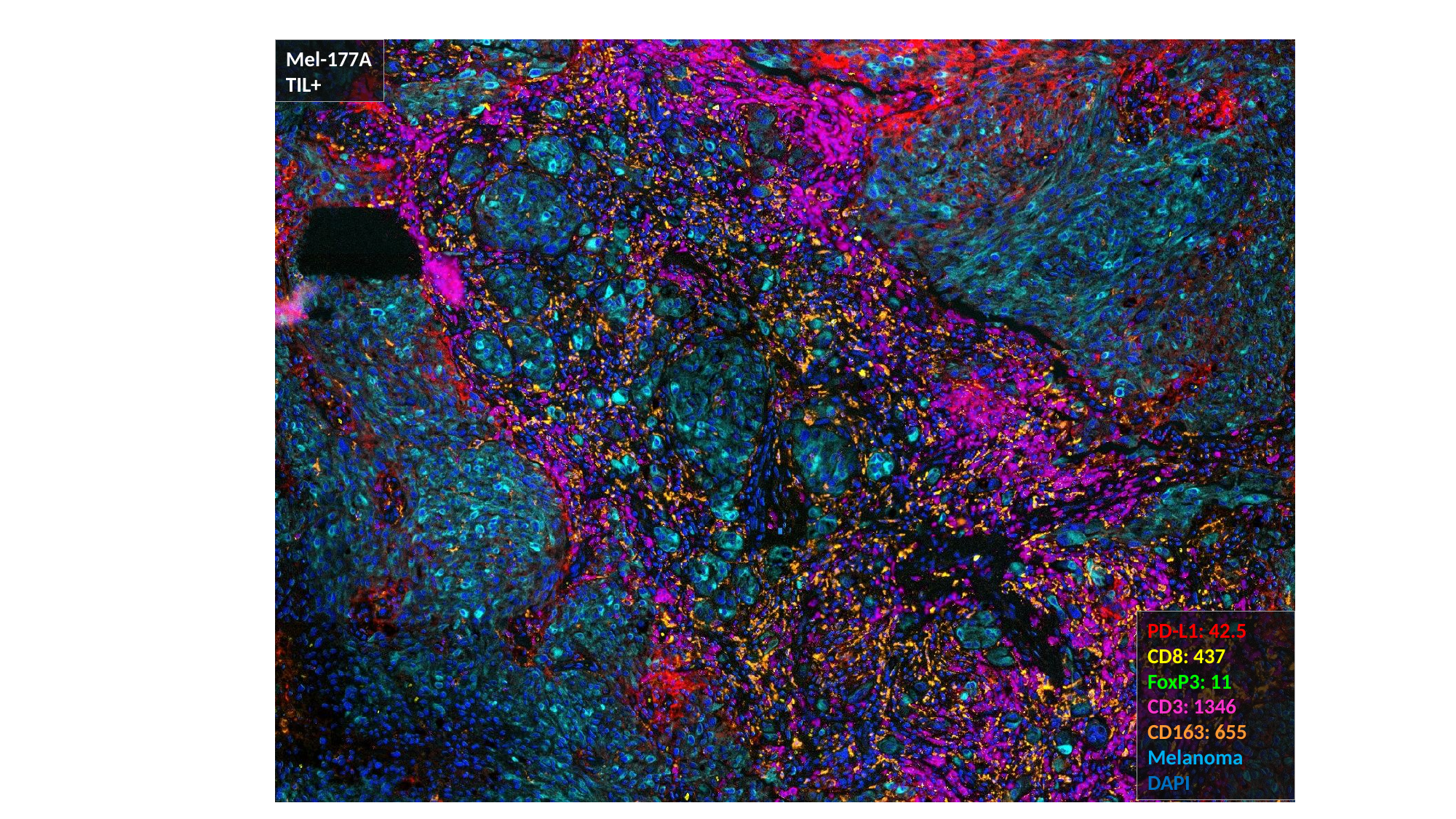

Figure S8
Mel-177A
TIL+
PD-L1: 42.5
CD8: 437
FoxP3: 11
CD3: 1346
CD163: 655
Melanoma
DAPI

## Slide 10
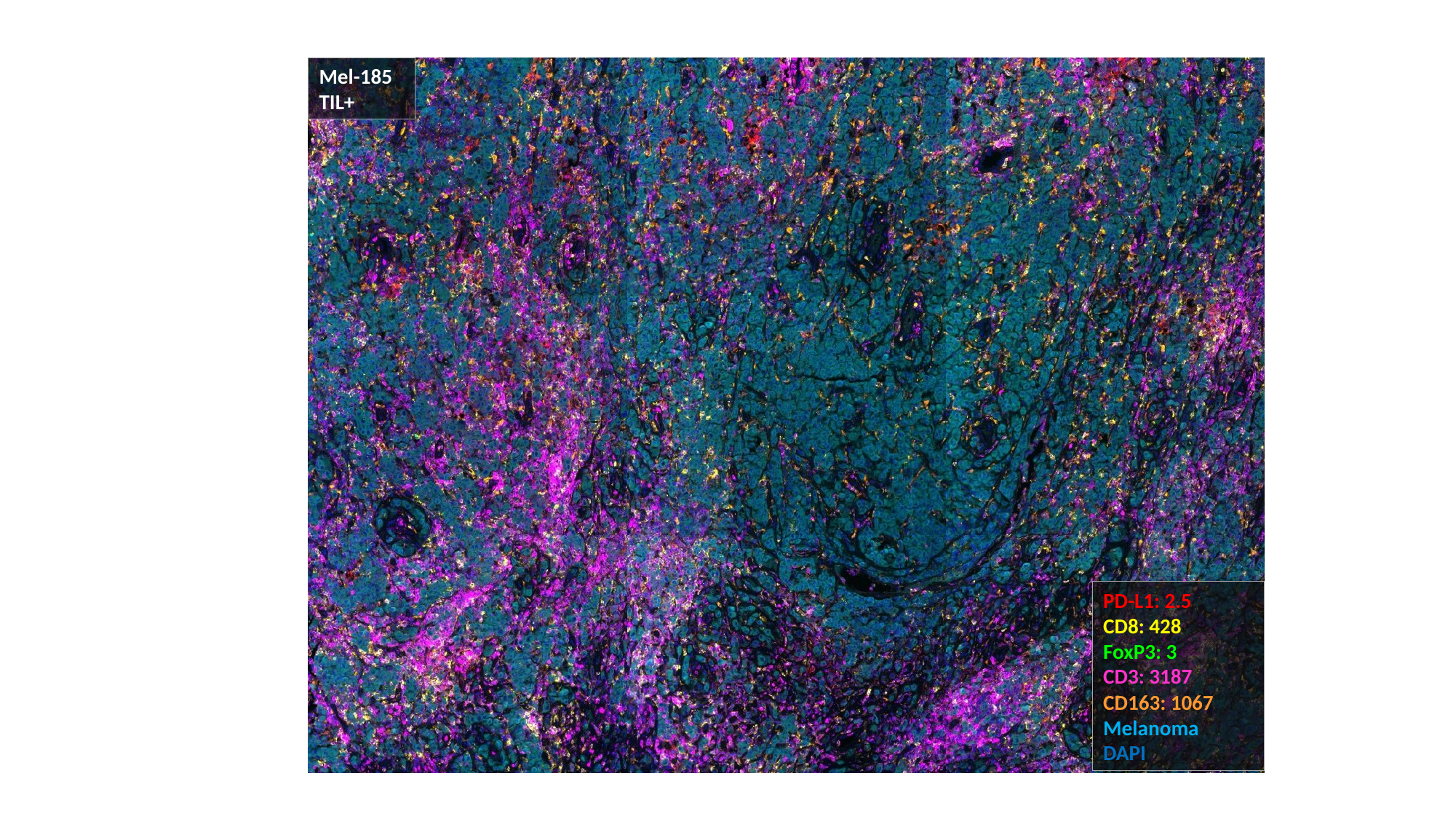

Figure S8
Mel-185
TIL+
PD-L1: 2.5
CD8: 428
FoxP3: 3
CD3: 3187
CD163: 1067
Melanoma
DAPI

## Slide 11
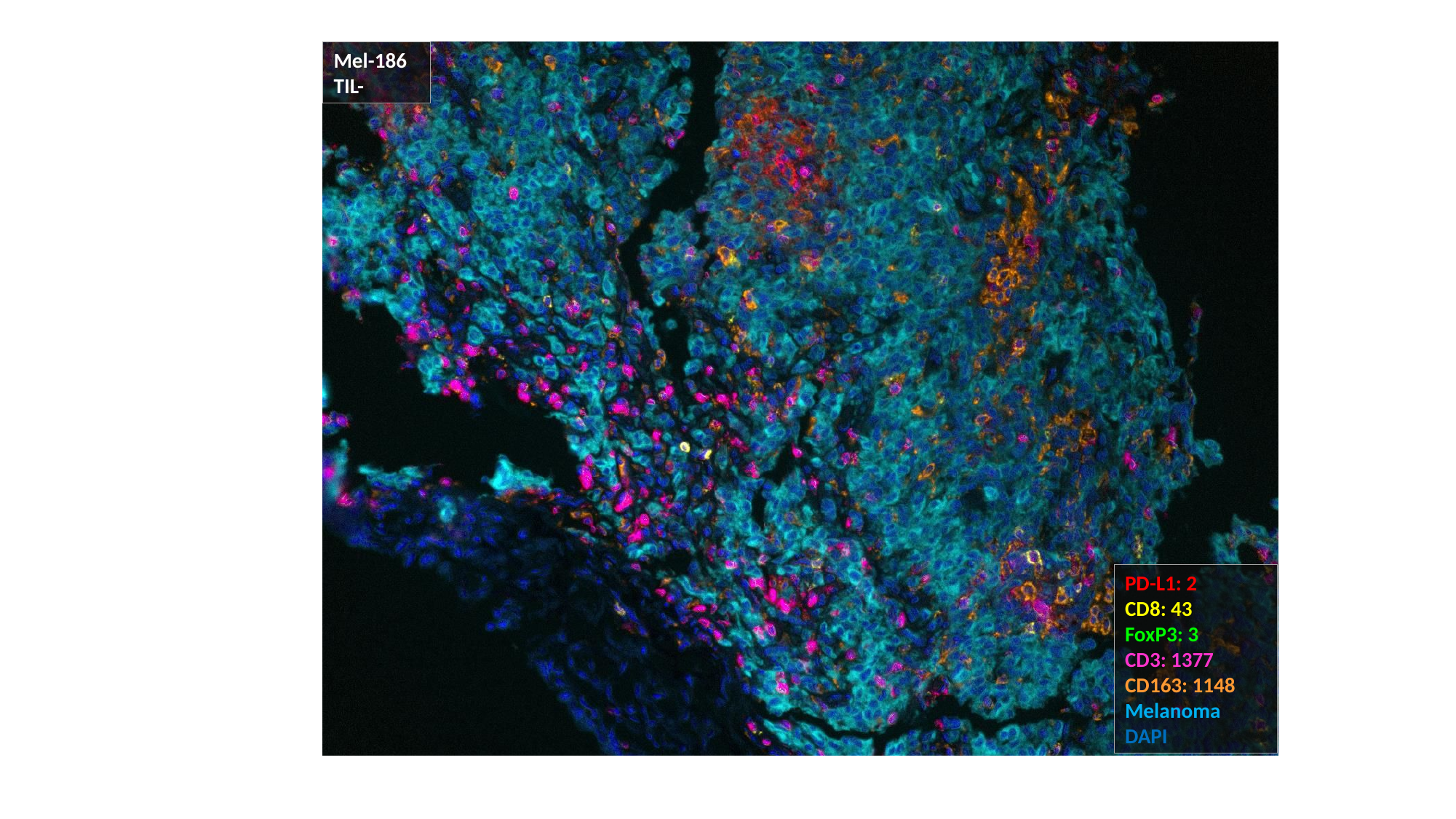

Figure S8
Mel-186
TIL-
PD-L1: 2
CD8: 43
FoxP3: 3
CD3: 1377
CD163: 1148
Melanoma
DAPI

## Slide 12
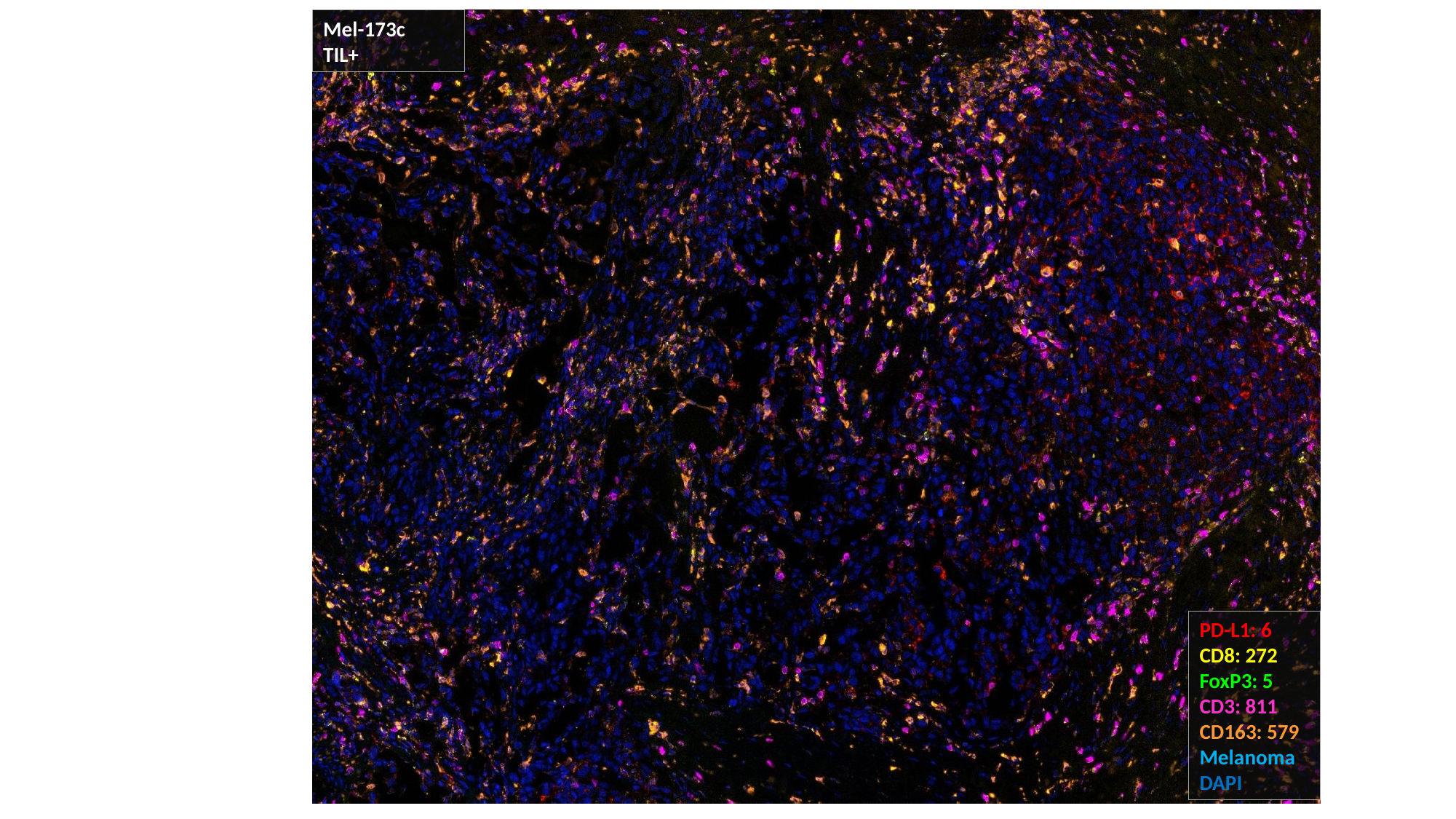

Figure S8
Mel-173c
TIL+
PD-L1: 6
CD8: 272
FoxP3: 5
CD3: 811
CD163: 579
Melanoma
DAPI

## Slide 13
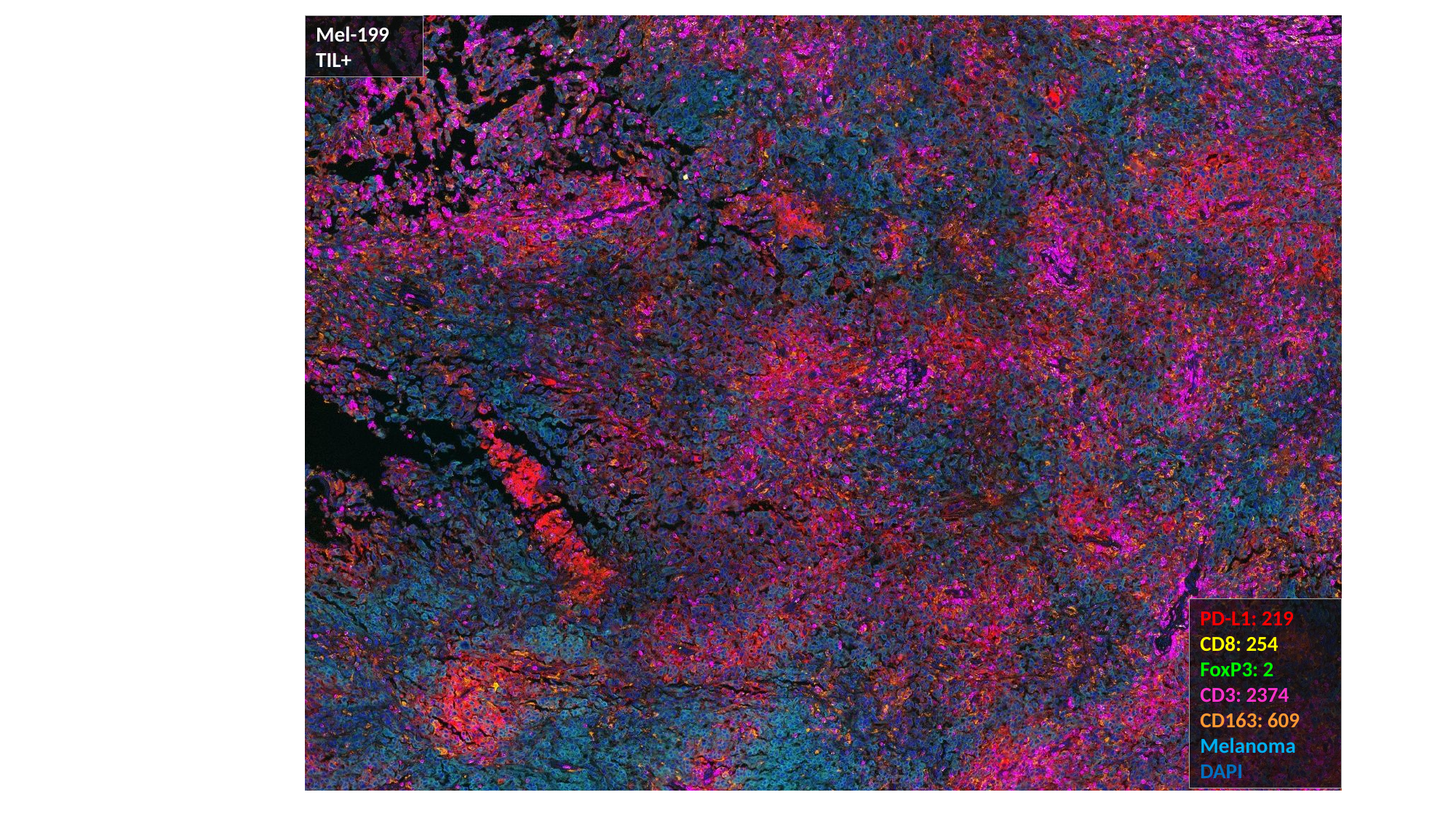

Figure S8
Mel-199
TIL+
PD-L1: 219
CD8: 254
FoxP3: 2
CD3: 2374
CD163: 609
Melanoma
DAPI

## Slide 14
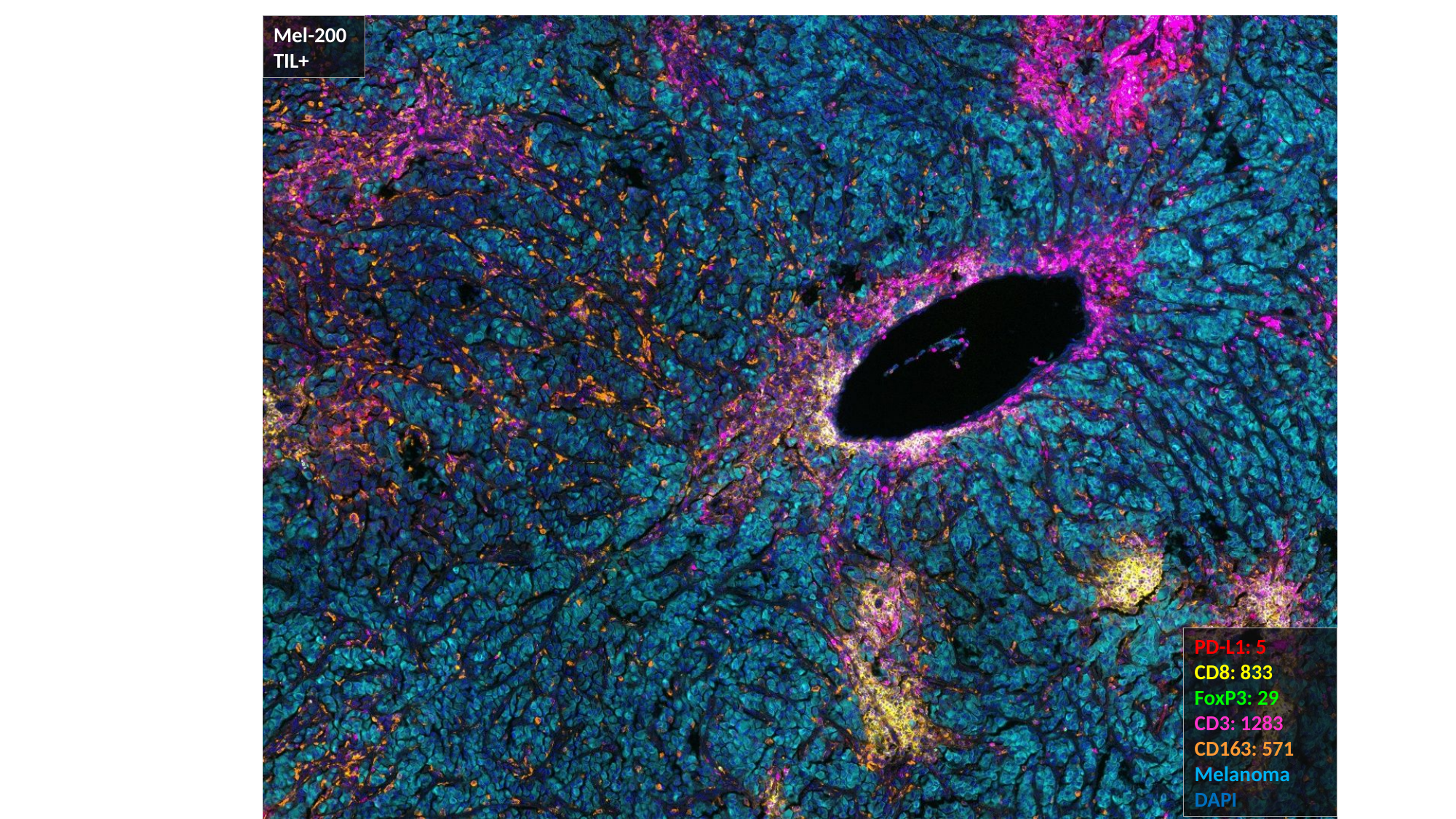

Figure S8
Mel-200
TIL+
PD-L1: 5
CD8: 833
FoxP3: 29
CD3: 1283
CD163: 571
Melanoma
DAPI

## Slide 15
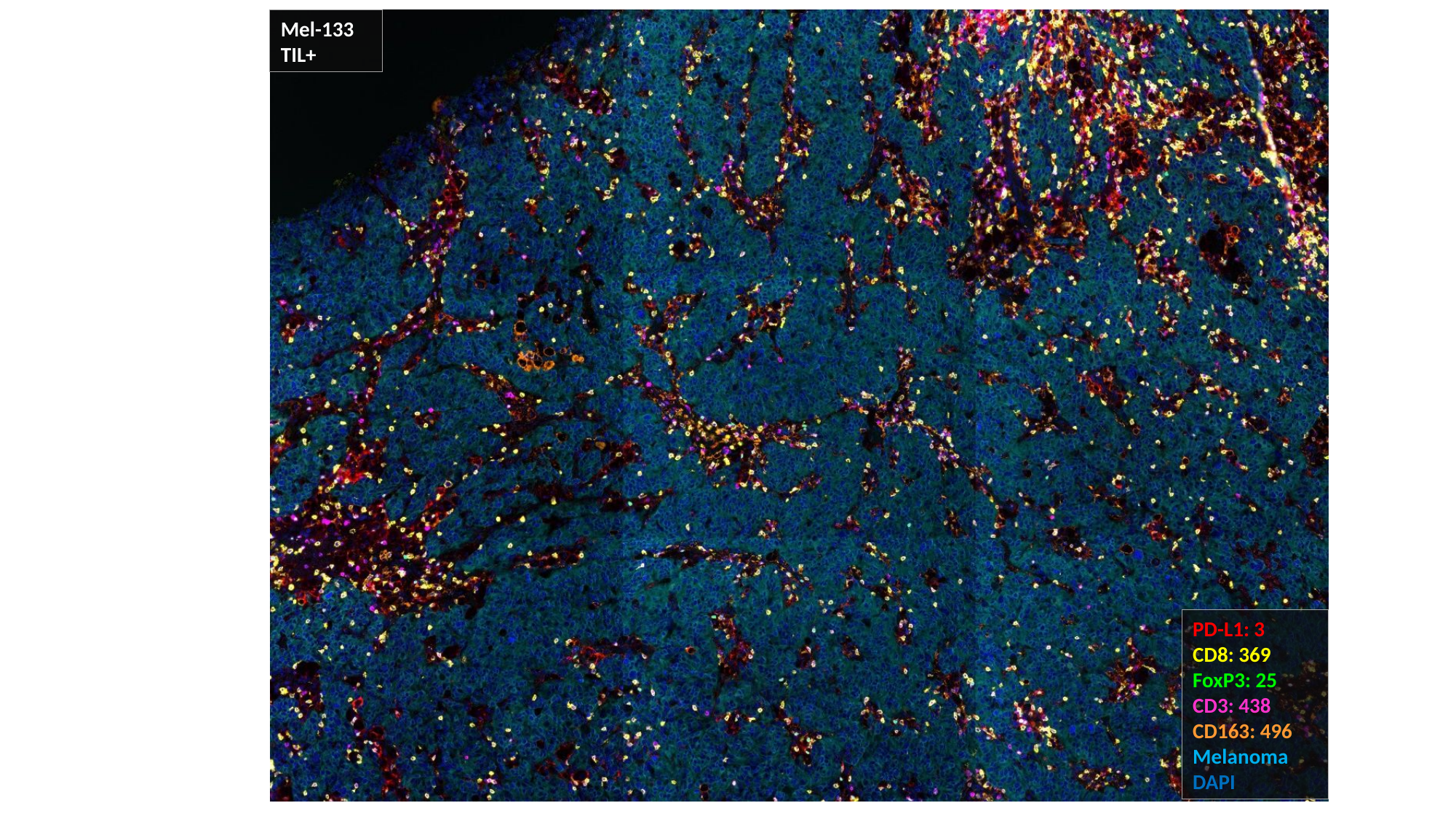

Figure S8
Mel-133
TIL+
PD-L1: 3
CD8: 369
FoxP3: 25
CD3: 438
CD163: 496
Melanoma
DAPI

## Slide 16
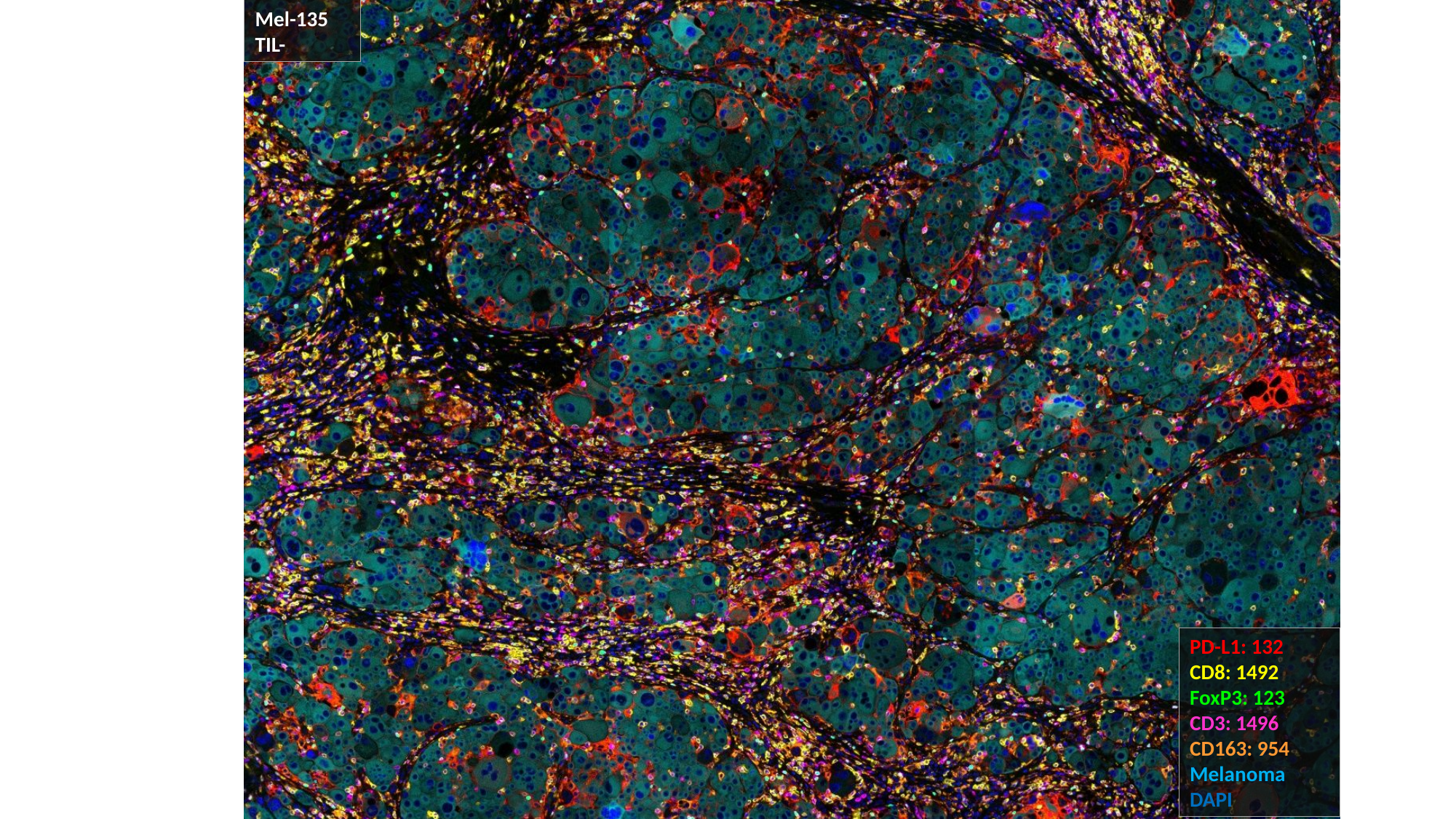

Mel-135
TIL-
Figure S8
PD-L1: 132
CD8: 1492
FoxP3: 123
CD3: 1496
CD163: 954
Melanoma
DAPI

## Slide 17
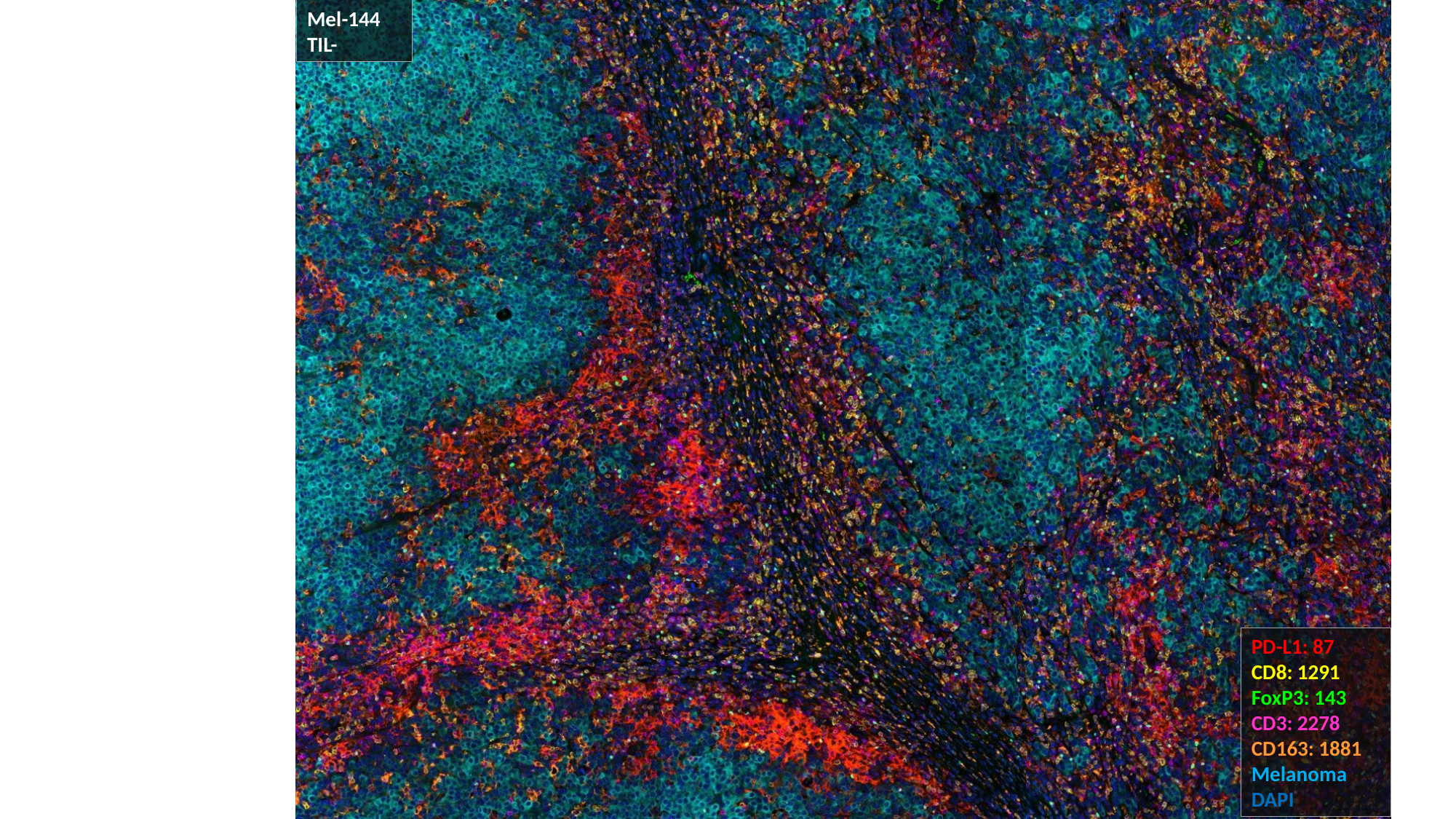

Mel-144
TIL-
Figure S8
PD-L1: 87
CD8: 1291
FoxP3: 143
CD3: 2278
CD163: 1881
Melanoma
DAPI

## Slide 18
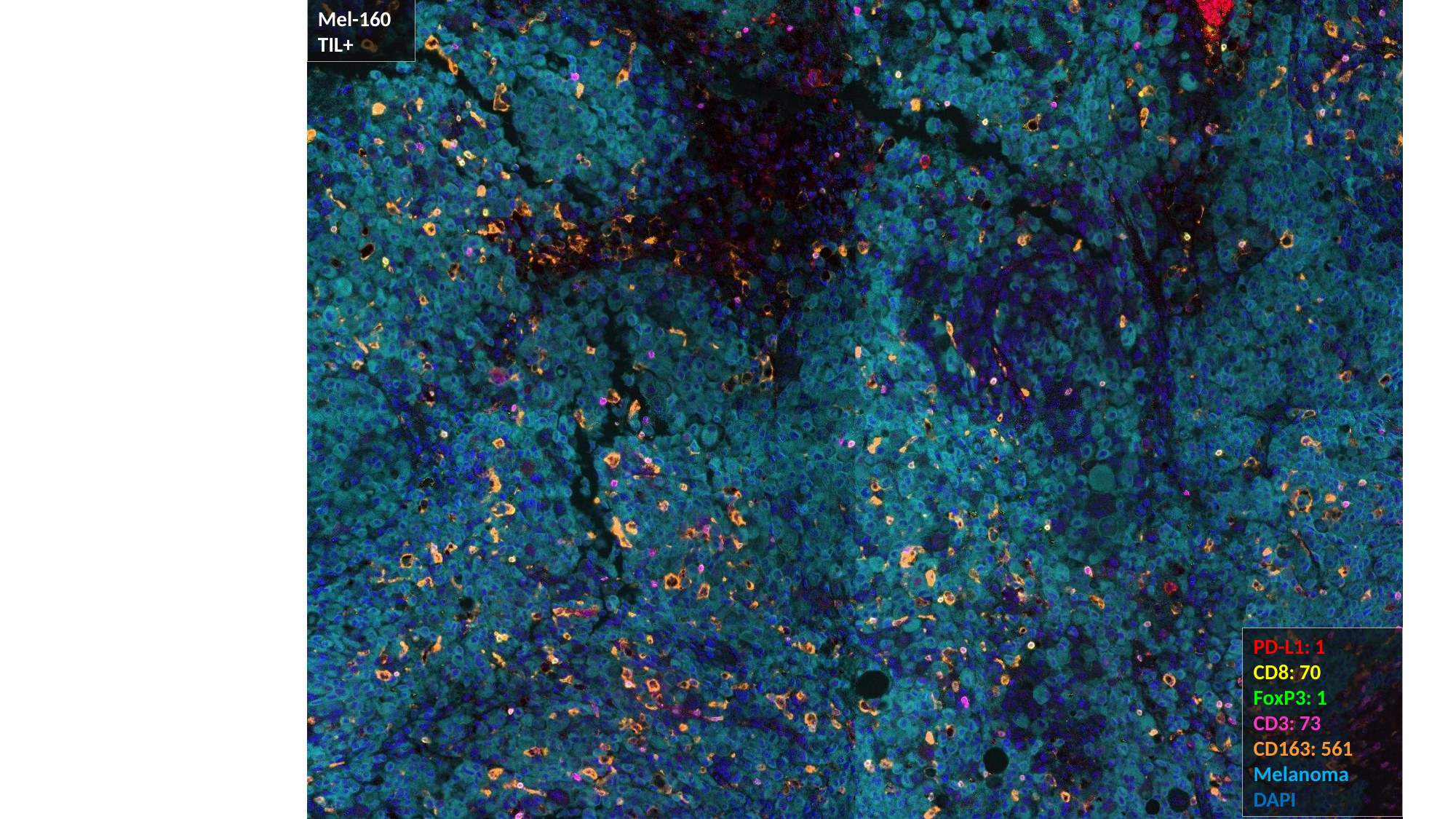

Mel-160
TIL+
Figure S8
PD-L1: 1
CD8: 70
FoxP3: 1
CD3: 73
CD163: 561
Melanoma
DAPI

## Slide 19
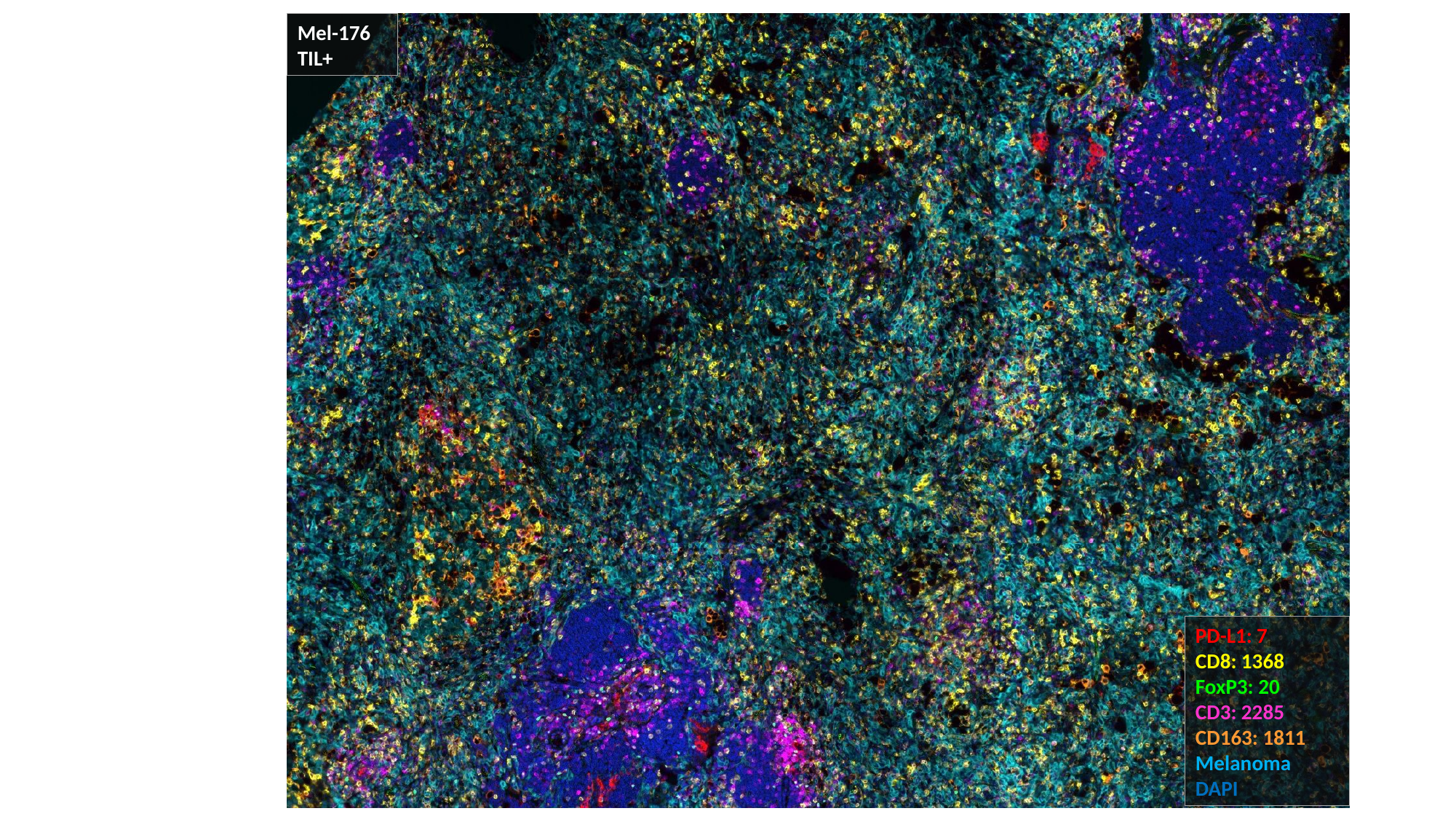

Figure S8
Mel-176
TIL+
PD-L1: 7
CD8: 1368
FoxP3: 20
CD3: 2285
CD163: 1811
Melanoma
DAPI

## Slide 20
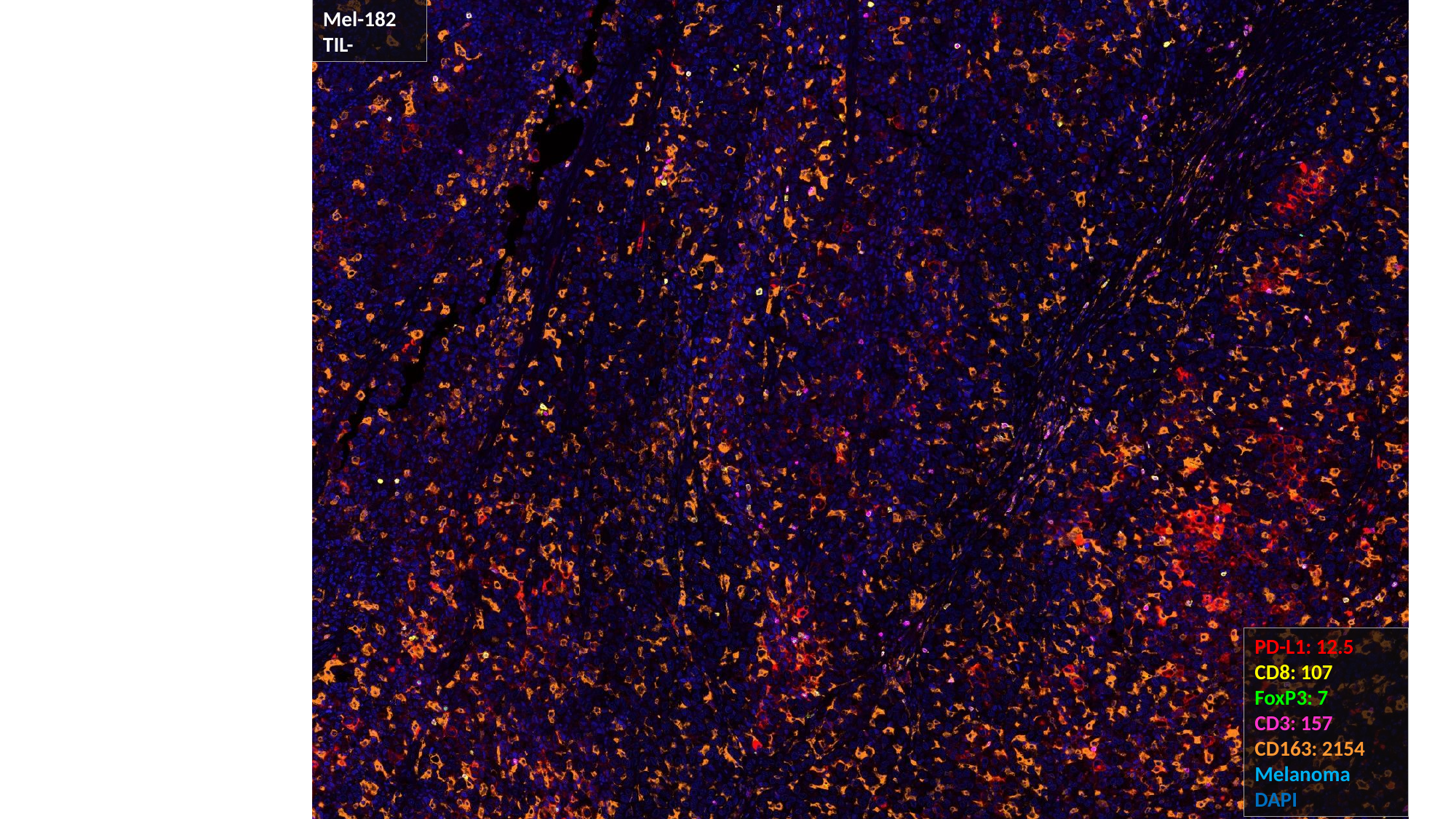

Mel-182
TIL-
Figure S8
PD-L1: 12.5
CD8: 107
FoxP3: 7
CD3: 157
CD163: 2154
Melanoma
DAPI

## Slide 21
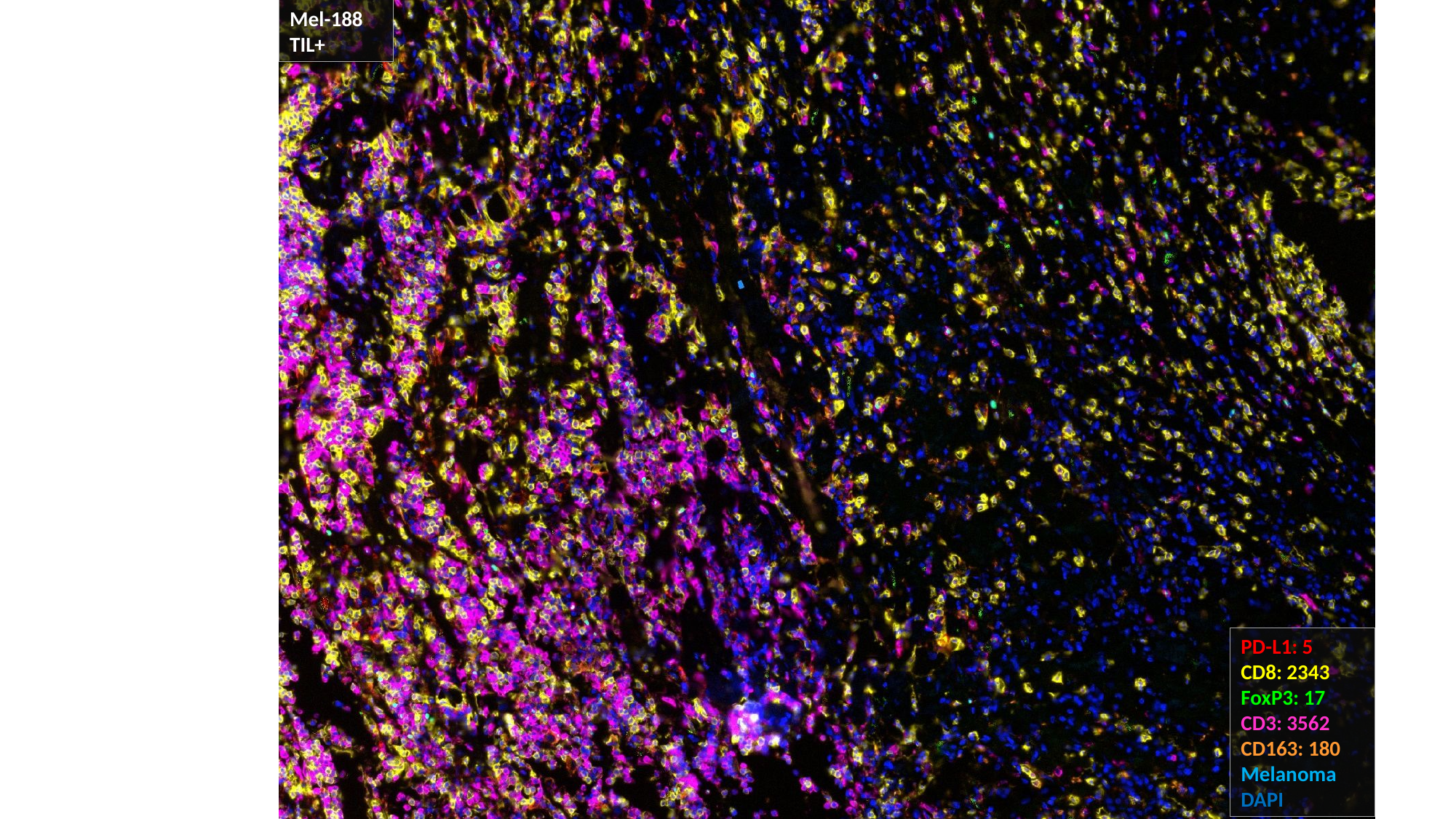

Mel-188
TIL+
Figure S8
PD-L1: 5
CD8: 2343
FoxP3: 17
CD3: 3562
CD163: 180
Melanoma
DAPI

## Slide 22
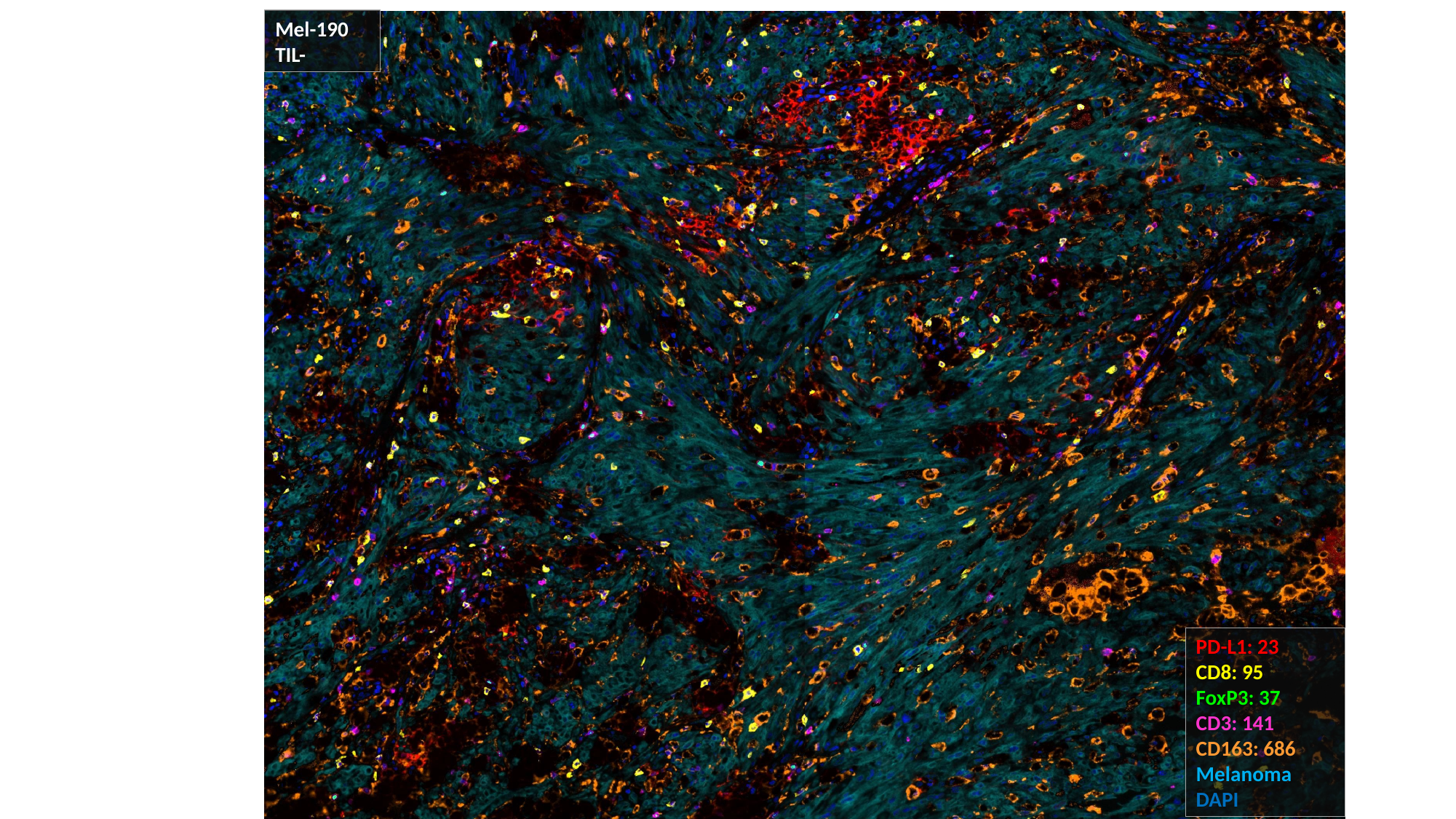

Figure S8
Mel-190
TIL-
PD-L1: 23
CD8: 95
FoxP3: 37
CD3: 141
CD163: 686
Melanoma
DAPI

## Slide 23
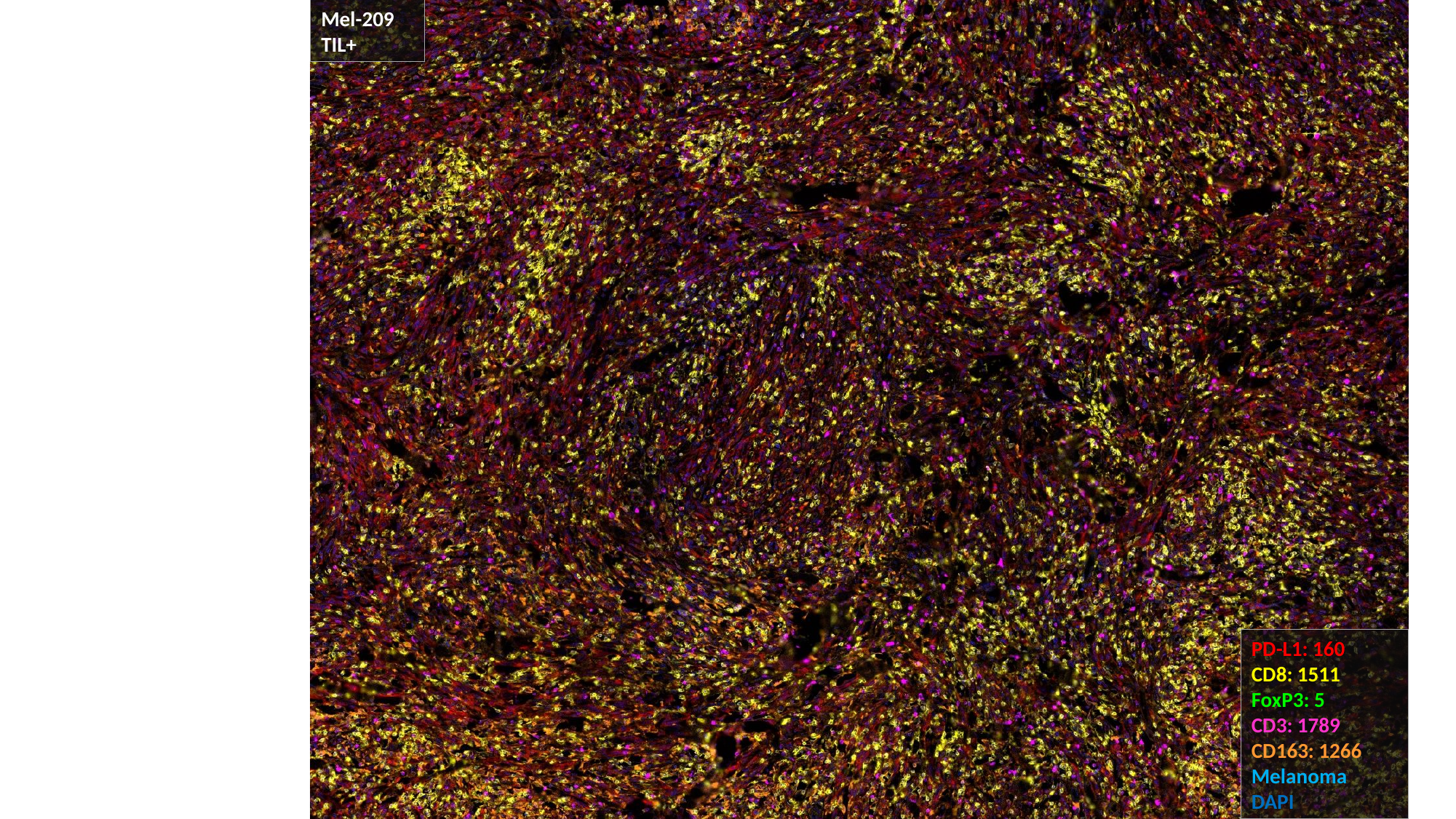

Mel-209
TIL+
Figure S8
PD-L1: 160
CD8: 1511
FoxP3: 5
CD3: 1789
CD163: 1266
Melanoma
DAPI

## Slide 24
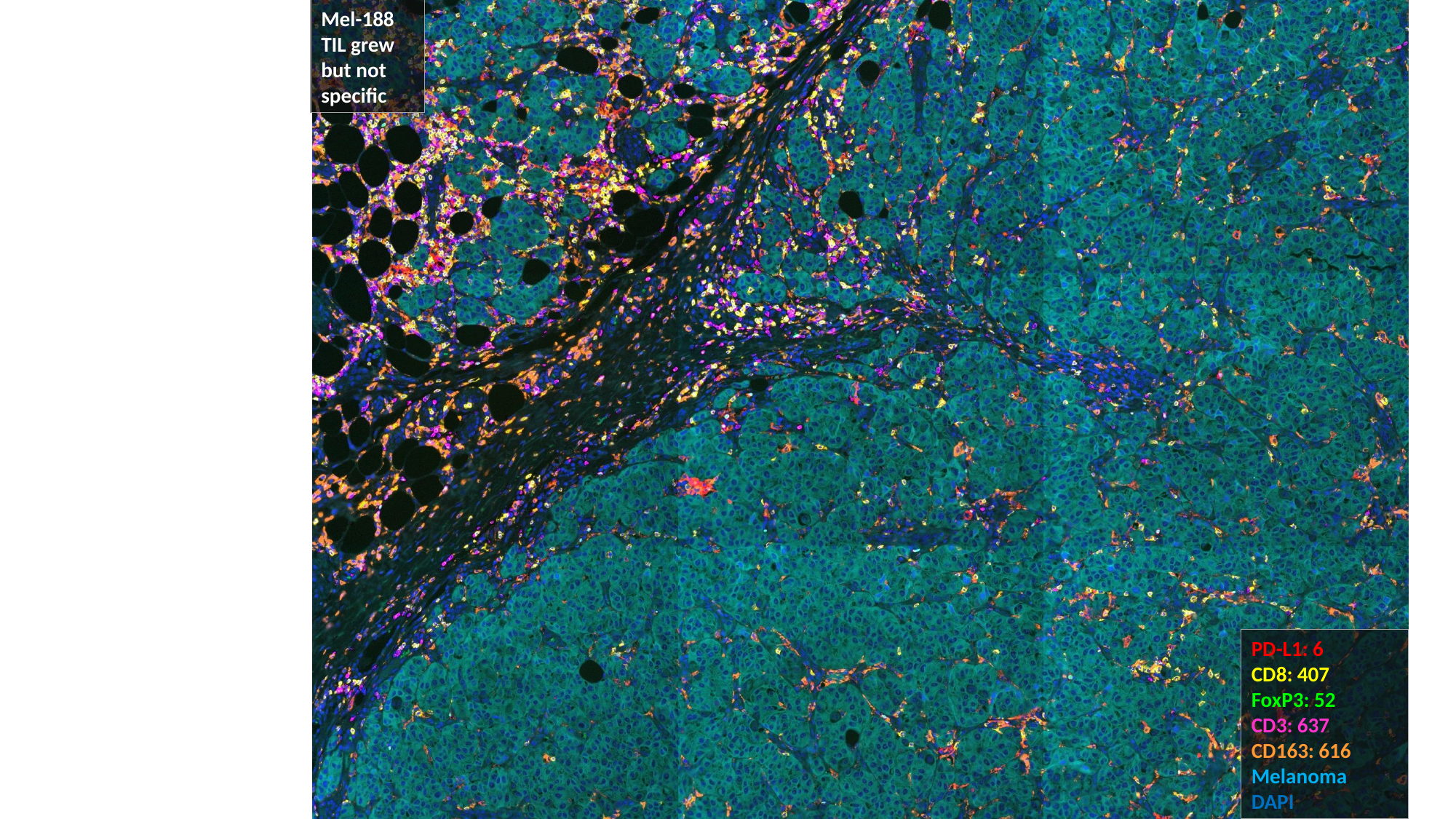

Mel-188
TIL grew but not specific
Figure S8
PD-L1: 6
CD8: 407
FoxP3: 52
CD3: 637
CD163: 616
Melanoma
DAPI

## Slide 25
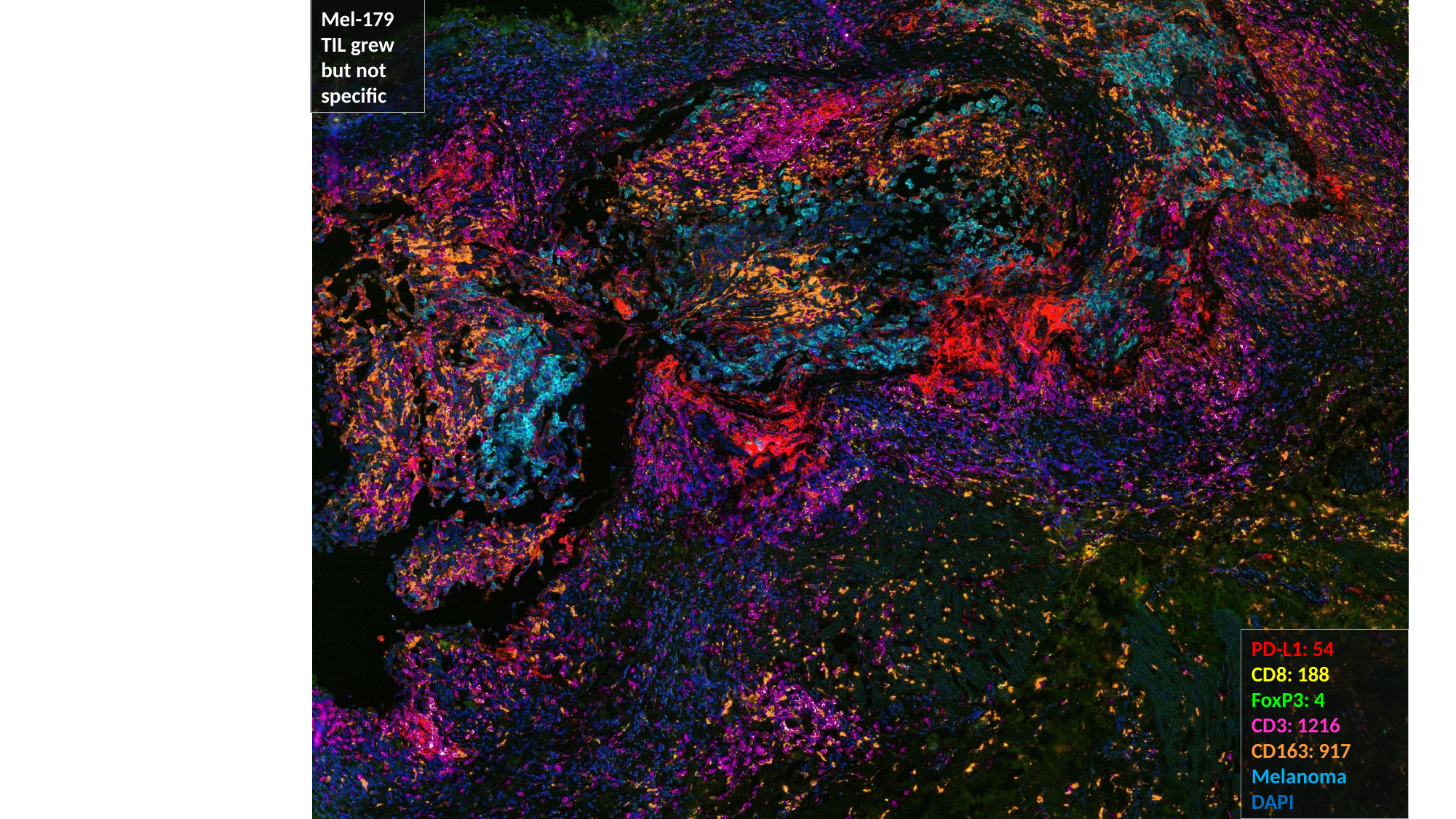

Mel-179
TIL grew but not specific
Figure S8
PD-L1: 54
CD8: 188
FoxP3: 4
CD3: 1216
CD163: 917
Melanoma
DAPI
