# Supplementary material for: Multispectral imaging of formalin-fixed tissue predicts ability to generate tumor-infiltrating lymphocytes from melanoma
Source: J Immunother Cancer. 2015 Oct 20;3:47. doi: 10.1186/s40425-015-0091-z (PMC4617712; doi:10.1186/s40425-015-0091-z)
Supplement: Additional file 2: — Supplemental methods. Detailed immunohistochemistry methods for multispectral analysis. (DOCX 20 kb) [file 40425_2015_91_MOESM2_ESM.docx]

**2. Supplemental Methods**

**2.1. Slide Preparation**

1. Formalin-fixed-paraffin-embedded (FFPE) tumor samples
2. Cardinal ColorFrost Plus Slides (M6148-3Y)
3. Thermo Microtome (HM-355S)
4. Slide Storage Box (100 slides, Fisher 03-446)
5. Fisher HealthCare™ Tissue Floatation Baths (22-047-773)

**2.2. Deparaffinization and Antigen Retrieval**

1. Leica Autostainer (HC-9.1)
2. Fisher Reagent grade mixed xylenes (X5P-1GAL)
3. Fisher Reagent grade 100% alcohol (A962F-1GAL)
4. Slide dishes and racks from VWR Tissue-Tek slide staining set (25608-902)
5. Biogenex 10X Citra antigen retrieval buffer (HK086-9K)
6. Panasonic 1250W Microwave with Inverter Technology (NN-H965BF)

**2.3. Antibody staining 6-Plex Panel**

1. Infors HT Labotron Orbital Shaker (S-000115640)
2. Newcomer Supply Slideshow 30 microscope staining tray (6844-30CL)
3. Vector Labs ImmEdge Hydrophobic Barrier Pen (H-4000)
4. Cell Signaling Technology rabbit anti-PD-L1 antibody (E1L3N, 13684)
5. Spring Biosciences rabbit anti-CD8 antibody (SP239, M5394)
6. Abcam mouse anti-FoxP3 antibody (236A/E7, AB20034)
7. Spring Biosciences rabbit anti-CD3 antibody (SP7, M3070)
8. Ventana mouse anti-CD163 antibody (MRQ-26, 760-4437)
9. Abcam mouse anti-melanoma antibody (HMB45/DT101/BC199/T311, ab733)
10. Life Technologies SuperPicture Polymer Detection Kit – Rabbit (87-9623)
11. Life Technologies SuperPicture Polymer Detection Kit – Mouse (87-9163)
12. PerkinElmer Opal 3-plex Kit (NEL791001KT)
13. PerkinElmer TSA Coumarin system (NEL703001KT)
14. PerkinElmer TSA Biotin (NEL700001KT)
15. Life Technologies TSA Reagent Alexa Fluor 594 Tyramide (T20950)
16. Life Technologies Alexa Fluor 514 Streptavidin (S32353)
17. Life Technologies DAPI (D-1306)
18. Ventana Antibody Diluent (251-018)
19. Sigma Trizma Base (251-018)
20. Sigma Sodium Chloride (S9888-500G)
21. Sigma Hydrochloric acid (258148-500ML)
22. Lonza 1X D-PBS (17-512F)
23. Vector Labs VectaShield Hardset Mounting Medium (H-1500)
24. Fast drying nail polish (L.A. colors, clear)
25. Vacuum source

**2.4. Imaging and Software Analysis**

1. PerkinElmer Vectra Microscope Bundle with Automated Slide Loader
2. PerkinElmer InForm Image Analysis software (Ver 2.1)

**3. Methods**

**3.1 Slide Preparation – 24 slides per run, 8 control slides and 16 test slides**

1. Retrieve FFPE tumor samples from pathology
2. Pre-chill samples on ice for 10 minutes and heat up water bath to 40°C
3. Slice 4µM thick ribbons using the microtome and float sections onto slides
4. Place slides in the box and let dry in room temperature overnight, store slides in 4°C until use.

**3.2 Deparaffinization and Antigen Retrieval**

1. Prepare 10X TBS solution by dissolving 88g of Trizma base and 24g of NaCl in ddH_2_O and adjust pH to 7.60 with 37% HCl (approximately 47.5mL for 1L 10X TBS)
2. Place slides onto staining rack in the Leica autostainer and run deparaffinization protocol. For the 6-plex panel, 8 single stain control slides are needed (6 for each fluorophor, 1 for DAPI and 1 unstained)
3. Mark the border of the tumor sample with ImmEdge hydrophobic pen and let slides dry for 5 minutes
4. Rinse the slides once with ddH_2_O and immerse the slides in 1X Citra antigen retrieval buffer
5. Place the lid, heat the dish for 1 minutes on 100% power, followed by 15 minutes on 10% power. Let the dish cool overnight.
6. Block the slides with Ventana antibody diluent for 10 minutes.
7. Dilute anti-PD-L1 antibody in Ventana antibody diluent at 1:200 concentration, add approximately 100µL per slide, covering the tissue area. Incubate for 30 minutes at room temperature (RT) shaking at 110 rpm.
8. Vacuum off the primary antibody, wash the slides 3x 30 seconds in 1X TBST (0.1% Tween).
9. Add anti-rabbit secondary antibody drop wise to cover the tissue area, incubate for 10 minutes at RT and 70 rpm.
10. Vacuum off secondary antibody, wash the slides 3x 30 seconds in 1X TBST.
11. Dissolve TSA-Cy5 from PerkinElmer Opal 3-Plex kit in 150µL DMSO, dilute 1:75 in Amplification *plus* buffer and add approximately 100µL per slide to cover the tissue area. Incubate for 10 minutes at RT and 110 rpm^1^. Store stock solution at 4°C.
12. Vacuum off TSA, wash the slides 3x 30 seconds in 1X TBST, followed by two rinses in ddH_2_O. Immerse slides in 1X Citra antigen retrieval solution.
13. Place the lid, heat the dish for 1 minutes on 100% power, followed by 12 minutes on 10% power. Let the dish cool for at least 15 minutes.
14. Block the slides with Ventana antibody diluent for 2 minutes.
15. Dilute anti-CD8 antibody in Ventana antibody diluent at 1:50 dilution, add approximately 100µL per slide, covering the tissue area. Incubate for 45 minutes at room temperature (RT) shaking at 110 rpm.
16. Vacuum off the primary antibody, wash the slides 3x 30 seconds in 1X TBST.
17. Add anti-rabbit secondary antibody drop wise to cover the tissue area, incubate for 10 minutes at RT and 70 rpm.
18. Vacuum off secondary antibody, wash the slides 3x 30 seconds in 1X TBST.
19. Dissolve TSA-Cy3 from PerkinElmer Opal 3-Plex kit in 150µL DMSO, dilute 1:75 in Amplification *plus* buffer and add approximately 100µL per slide to cover the tissue area. Incubate for 10 minutes at RT and 110 rpm. Store stock solution at 4°C.
20. Vacuum off TSA, wash the slides 3x 30 seconds in 1X TBST, followed by two rinses in ddH_2_O. Immerse slides in 1X Citra antigen retrieval solution.
21. Place the lid, heat the dish for 1 minutes on 100% power, followed by 12 minutes on 10% power. Let the dish cool for at least 15 minutes.
22. Block the slides with Ventana antibody diluent for 2 minutes.
23. Dilute anti-FoxP3 antibody in Ventana antibody diluent at 1:100 dilution, add approximately 100µL per slide, covering the tissue area. Incubate for 1 hour at room temperature (RT) shaking at 110 rpm.
24. Vacuum off the primary antibody, wash the slides 3x 30 seconds in 1X TBST.
25. Add anti-mouse secondary antibody drop wise to cover the tissue area, incubate for 10 minutes at RT and 110 rpm.
26. Vacuum off secondary antibody, wash the slides 3x 30 seconds in 1X TBST.
27. Dissolve TSA-FITC from PerkinElmer Opal 3-Plex kit in 150µL DMSO, dilute 1:75 in Amplification *plus* buffer and add approximately 100µL per slide to cover the tissue area. Incubate for 10 minutes at RT and 110 rpm. Store stock solution at 4°C.
28. Vacuum off TSA, wash the slides 3x 30 seconds in 1X TBST, followed by two rinses in ddH_2_O. Immerse slides in 1X Citra antigen retrieval solution.
29. Place the lid, heat the dish for 1 minutes on 100% power, followed by 12 minutes on 10% power. Let the dish cool for at least 15 minutes.
30. Block the slides with Ventana antibody diluent for 2 minutes.
31. Dilute anti-CD3 antibody in Ventana antibody diluent at 1:50 dilution, add approximately 100µL per slide, covering the tissue area. Incubate for 45 minutes at room temperature (RT) shaking at 110 rpm.
32. Vacuum off the primary antibody, wash the slides 3x 30 seconds in 1X TBST.
33. Add anti-rabbit secondary antibody drop wise to cover the tissue area, incubate for 10 minutes at RT and 70 rpm.
34. Vacuum off secondary antibody, wash the slides 3x 30 seconds in 1X TBST.
35. Dissolve TSA-AF594 from Life Technologies in 150µL DMSO, dilute 1:75 in Amplification buffer^2^ and add approximately 100µL per slide to cover the tissue area. Incubate for 10 minutes at RT and 110 rpm. Store stock solution at 4°C.
36. Vacuum off TSA, wash the slides 3x 30 seconds in 1X TBST, followed by single rinse in ddH_2_O. Immerse slides in 1X Citra antigen retrieval solution.
37. Place the lid, heat the dish for 1 minutes on 100% power, followed by 12 minutes on 10% power. Let the dish cool for at least 15 minutes.
38. Block the slides with Ventana antibody diluent for 2 minutes.
39. Add approximately 100µL of anti-CD163 prediluted antibody per slide, covering the tissue area. Incubate for 30 minutes at room temperature (RT) shaking at 110 rpm.
40. Vacuum off the primary antibody, wash the slides 3x 30 seconds in 1X TBST.
41. Add anti-mouse secondary antibody drop wise to cover the tissue area, incubate for 10 minutes at RT and 70 rpm.
42. Vacuum off secondary antibody, wash the slides 3x 30 seconds in 1X TBST.
43. Dissolve TSA-Biotin from PerkinElmer 1200µL DMSO, dilute 1:75 in Amplification buffer and add approximately 100µL per slide to cover the tissue area. Incubate for 10 minutes at RT and 110 rpm. Store stock solution at 4°C.
44. Vacuum off TSA, wash the slides 3x 30 seconds in 1X TBST, followed by single rinse in ddH_2_O. Immerse slides in 1X Citra antigen retrieval solution.
45. Place the lid, heat the dish for 1 minutes on 100% power, followed by 12 minutes on 10% power. Let the dish cool for at least 15 minutes.
46. Block the slides with Ventana antibody diluent for 2 minutes.
47. Dilute anti-Melanoma antibody in Ventana antibody diluent at 1:25 dilution, add approximately 100µL per slide, covering the tissue area. Incubate for 1 hour at room temperature (RT) shaking at 110 rpm.
48. Vacuum off the primary antibody, wash the slides 3x 30 seconds in 1X TBST.
49. Add anti-mouse secondary antibody drop wise to cover the tissue area, incubate for 10 minutes at RT and 70 rpm.
50. Vacuum off secondary antibody, wash the slides 3x 30 seconds in 1X TBST.
51. Dissolve TSA-Coumarin from PerkinElmer in 600µL DMSO, dilute 1:75 in Amplification buffer and add approximately 100µL per slide to cover the tissue area. Incubate for 10 minutes at RT and 110 rpm. Store stock solution at 4°C
52. Vacuum off TSA, wash the slides 3x 30 seconds in 1X TBST
53. Dissolve Life Technologies Streptavidin-AF514 (1mg/mL stock) 1:50 in D-PBS and add 100µL per slide to cover the tissue area. Incubate for 10 minutes at RT and 110 rpm. Store stock solution at 4°C
54. Vacuum off AF514, wash the slides 2x 30 seconds in 1X TBST
55. Dilute DAPI (1mg/mL stock) 1:500 in D-PBS and add 100µL per slide to cover the tissue area. Incubate for 5 minutes at RT and 110 rpm. Store stock solution at 4°C
56. Vacuum off DAPI, wash the slides 2x 30 seconds in 1X TBST
57. Rinse slides once with ddH_2_O
58. Coverslip the slides with VectraShield Hard Mount
59. Paint the edge of the slide with nail polish and store slides at 4°C in a covered slide box

**3.4 Imaging and Software Analysis**

1. Load a slide onto PerkinElmer Vectra Microscope and open Vectra software
2. Create new protocol with user defined regions
3. Adjust exposure for each channel and use the same exposure for the experiment
4. Select two to three 3x3 (18-27) representative regions of interest for high powered (200x) imaging
5. Load up single color control slides and take single 200x image for each single color control and unstained using the same exposure settings
6. Open InForm and build single color compensation library using the single color control slides
7. Open a few representative multispectral images from the experiment, unmix channels using the single color compensation library and follow tissue segmentation^3^, cell segmentation, and scoring.
8. Batch process all remaining multispectral images from the experiment
9. Merge and process data in Excel and perform relevant statistical analysis in Prism

**Notes**

1. Do not exceed 10 minutes, as background can increase significantly. When having multiple slides, take into account the time it takes between adding solution to the first slide and the last slide (i.e. if it takes 2 minutes to add reagents to all slides, incubate for additional 8 minutes).
2. Life Technologies TSA-AF594 comes with supplied amplification buffer. However for best results use PerkinElmer Amplification *Plus* Buffer.
3. The melanoma cocktail (Melan-A, GP100, HMG45) is only positive in 70% of the analyzed melanoma tumors in our cohort. Thus for tissue segmentation to separate tumor from stroma, it is important to include plenty sample images from tumors that are negative for melanoma cocktail and include DAPI in addition to TSA-coumarin (fluorophore for melanoma cocktail).
